# Dissipation Function: Nonequilibrium Physics and Dynamical Systems

**DOI:** 10.3390/e22080835

**Published:** 2020-07-30

**Authors:** Salvatore Caruso, Claudio Giberti, Lamberto Rondoni

**Affiliations:** 1 Department of Physics, Informatics and Mathematics, Università di Modena and Reggio Emilia, via G.Campi 213/b, I-41125 Modena, Italy; 236561@studenti.unimore.it; 2 Department of Sciences and Methods for Engineering, Università di Modena and Reggio Emilia, via G.Amendola 2, I-42122 Reggio Emilia, Italy; claudio.giberti@unimore.it; 3 Department of Mathematical Sciences Politecnico di Torino, Corso Duca degli Abruzzi 24, I-10129 Torino, Italy; 4 INFN, Sezione di Torino, Via Pietro Giuria 1, 10125 Torino, Italy

**Keywords:** dissipative systems, entropy production, response theory, nonequilibrium steady states, nonequilibrium molecular dynamics, formal thermodynamics

## Abstract

An exact response theory has recently been developed within the field of Nonequilibrium Molecular Dynamics. Its main ingredient is known as the Dissipation Function, Ω. This quantity determines nonequilbrium properties like thermodynamic potentials do with equilibrium states. In particular, Ω can be used to determine the exact response of particle systems obeying classical mechanical laws, subjected to perturbations of arbitrary size. Under certain conditions, it can also be used to express the response of a single system, in contrast to the standard response theory, which concerns ensembles of identical systems. The dimensions of Ω are those of a rate, hence Ω can be associated with the entropy production rate, provided local thermodynamic equilibrium holds. When this is not the case for a particle system, or generic dynamical systems are considered, Ω can equally be defined, and it yields formal, thermodynamic-like, relations. While such relations may have no physical content, they may still constitute interesting characterizations of the relevant dynamics. Moreover, such a formal approach turns physically relevant, because it allows a deeper analysis of Ω and of response theory than possible in case of fully fledged physical models. Here, we investigate the relation between linear and exact response, pointing out conditions for the validity of the response theory, as well as difficulties and opportunities for the physical interpretation of certain formal results.

## 1. Introduction

Since the 1980s, a dynamical systems approach to nonequilibrium phenomena has been developed, side by side with the increase of computing power and the ability to perform Molecular Dynamics (MD) simulations. Classical MD consists of algorithms meant to solve Newton’s dynamical equations for interacting particle systems, that may be subjected to external fields. Such investigations result quite informative and practically useful, when quantum effects are irrelevant or negligible, which is the case of fluids under standard conditions.

Provided no dissipative forces are present, one speaks of equilibrium MD, which is widely used together with statistical mechanical relations, in order to compute: rheological properties, polymers behaviours in porous media, defects in crystals, friction between surfaces, formation of atomic clusters, features of biological macromolecules, formation of drugs, etc. The results are most satisfactory, and, in practice, difficulties only arise if the interatomic forces are too complicated to properly implement, if the required number of simulated particles is too large, or the simulation times too long. In fact, the statistical relations connecting microscopic dynamical quantities to macroscopic properties provide quite an accurate and complete description of material objects. Therefore, MD is used to properly interpret results of experiments, and even in place of expensive or impossible experiments. For instance, MD may illustrate the formation of crystal defects, the propagation of fracture fronts inside solids, and the thermal dilation of nuclear fuel pellets inside a nuclear reactor, just to mention a few examples. The relevant literature is enormous, and dates back to early days of electronic computers, the Fermi–Pasta–Ulam–Tsingou problem being a forerunner that opened the way to an incredible number of mathematical, computational and physical advances [1,2]; see e.g., the books [3,4,5,6,7].

When the system of interest is driven away from equilibrium by external forces that continually feed energy in it and, at the same time, it is in contact with some environment that removes energy from it, a nonequilibrium steady state may be reached. The question is how to effectively model the typically very large environment [8]. Various methods have been developed for that. In particular, researchers introduced “synthetic forces” meant to efficiently represent the effect of a bath or reservoir on the property of interest for the system of interest, without simulating the atoms of the environment. Nonequilibrium MD (NEMD) was thus born, focusing on specific quantities of specific systems [6,9].

The term “synthetic” means that such forces do not exist in nature; they merely constitute a *“mathematical device used to transform a difficult boundary condition problem, the flow of heat in a system bounded by walls maintained at differing temperatures, into a much simpler mechanical problem”*, cf. the Introduction of Ref. [6]. In practice, the synthetic forces constrain in various ways the dynamics of particle systems fixing, for instance, the average of the fluctuating kinetic temperature. Such an approach is so successful that the corresponding algorithms are now provided as default routines of MD packages [10,11].

If the constraints are holonomic and the other forces conservative, the resulting equations of motion are Hamiltonian [12]; otherwise they are non-Hamiltonian and, in particular, they are dissipative, in the sense that on average they contract phase space volumes, although they remain time reversal invariant [6]. Consequently, from the angle of dynamical systems, their invariant measures are singular with respect to the Lebesgue measure. This approach may sound odd, if one expects all features of the dynamics to be derived from a Hamiltonian, but it simply replicates the usual construction of effective models, that bridge the gap between different levels of descriptions of natural phenomena, [13]. For instance, the Boltzmann equation and the Langevin equation alter the Hamiltonian dynamics to the point that it becomes irreversible and not deterministic; at the same time their success and foundational importance is not questionable. Justifications of the NEMD approach, within its specific realm, have been provided, demonstrating, for instance, that it properly computes transport coefficients [6]. Moreover, NEMD has been shown to provide a route to extend to dissipative dynamics the theory of equivalence of ensembles, as well as Khinchin’s approach to ergodic theory in physics [6,9,14,15,16].

The point is the following: no mathematical model spans all space and time scales of interest for a given material object: a model is acceptable when it describes a certain set of properties that are observable within given space and time scales. Of course, the wider the range of applicability of the model, the better it is. In this respect, classical Hamiltonian dynamics is no exception. It constitutes a tremendously effective microscopic model for a vast variety of non-dissipative phenomena, but dissipation makes its use problematic, cf. recent investigations on kinetic theory [17,18]. Analogously, models derived within the framework of NEMD, such as Gaussian thermostatted dynamics, or Nosé–Hoover thermostatted dynamics can be accepted as valid models of certain phenomena. The question is how many aspects of a given phenomenon are properly described by NEMD models. As generally true in physics, interesting results and relations can be derived in uncharted territories, but only experience can eventually confirm or refute their validity.

As a matter of fact, NEMD has become popular for its effectiveness in computing transport coefficients, such as the viscosity of a fluid [6]. It was also realized that such coefficients can be obtained from dynamical systems properties such as the sum of Lyapunov exponents or, under special conditions, just from the sum of the largest and of the smallest exponent [6]. In turn, −Λ, the opposite of the phase space volumes variation rate Λ, which turns out to be proportional to the thermostatting (fictitious or synthetic) term may also be proportional to the entropy production σ when thermodynamics applies, see e.g., Ref. [19]. (A similar idea had previously been independently investigated by Andrej, who followed a different approach [20]). This idea became very popular when Evans, Cohen and Morriss applied a representation of SRB measures to the fluctuations of Λ in a Gaussian isoenergetic shearing system [21], as in that case such a quantity equals the entropy production, when local thermodynamic equilibrium (LTE) holds. That paper derived and tested the Fluctuation Relation (FR), one of the rare exact relations for nonequilibrium thermodynamics based on microscopic deterministic dynamics. For a time reversal invariant model of shearing fluids, the FR states that positive values of the energy dissipation, are exponentially more probable than their opposite. This was interpreted as an explanation of the second law of thermodynamics, and motivated a flurry of activity. Moreover, it led various authors to identify the entropy production with the phase space contraction [22,23].

Although Λ is relatively easy to handle in dynamical systems theory, it was pointed out that its identification with σ is only justified in a limited set of cases [24,25]. Furthermore, in time dependent settings and in presence of fluctuations, the connection of Λ with physically measurable properties results rather loose. It was then shown that the physically relevant quantity in the cases described by NEMD is the so called Dissipation Function Ω, which preserves the meaning of energy dissipation rate even when LTE is violated, and thermodynamic quantities such as σ do not exist. Only in a few cases Ω equals −Λ, although steady state averages of Ω and −Λ may be simply related to each other.

Later Ω became the basis for a general response theory, in which it plays a role as fundamental for nonequilibrium states as thermodynamic potentials do for equilibrium states. It can be used to solve in terms of physically relevant quantities the Liouville equation and, consequently, to investigate the exact average response of the observables of ensembles of systems. Furthermore, Ω can be used to provide conditions for the evolution of single systems, [26,27]. (This should be contrasted with the fact that almost invariably response of systems to perturbations is given in terms of ensembles, and that a comparatively limited amount of works have tackled response beyond its linear approximations). Most importantly, these results are expressed in terms of a physically measurable quantity, rather than in terms of an abstract phase space quantity.

Unfortunately, it is rather complicated to thoroughly investigate the properties of the dissipation function in realistic particle models. Therefore, we consider mathematically amenable dynamical systems, even if their physical relevance may be limited, as often and successfully done in statistical physics, with the purpose of illustrating aspects of the relation between dynamics and thermodynamics; think for example of Helmoltz’s celebrated theorem [28]. Recently, Qian, Wang and Yi have also followed this route, scrutinizing a number of interesting examples and investigating them in terms of the phase space contraction rate −Λ [29]. The result is a set of intriguing relations between different dynamical quantities and formal thermodynamic quantities. In consideration of the fact that small systems, hence non-thermodynamic fluctuating quantities, as well as time dependent situations are ever more relevant in science and technology, we are going to perform a similar analysis within the dissipation function formalism.

This paper is organized as follows. Section 2 summarizes the theory of the Dissipation Function, also analyzing exactly solvable examples and numerical simulations. Section 3 compares linear Green–Kubo and exact response. Section 4 follows in part Ref. [29], using Ω rather than Λ. The two approaches appear as largely equivalent, since they both arise from the solution of the Liouville equation. Section 5 explicitly solves the cases of simple attractors, and of small dissipation, in the sense of small Λ. Section 6 discusses with examples the conditions under which the exact response theory based on Ω holds, showing that Ω has to be continuous at least, and that the initial phase space probability density $f_{0}$ has to be positive in all the space that can be explored by the dynamics. This condition, called ergodic consistency [30] is reminiscent, but weaker than the requirement that initial and asymptotic measure be absolutely continuous with respect to each other, also called equivalence [31]. If that is not the case, modifications to the response formulae are required [32].

Finally, Section 7 summarises our work, highlighting two points in particular: investigating simple systems allows us to understand properties of Ω that are not immediately evident in realistic particle systems; on the other hand we propose a kind of thermodynamic formalism for the analysis of dynamical systems, cf. Ref. [33], that is based on the language of nonequilibrium, rather than of equilibrium, statistical mechanics. Section 7 also stresses the role of Ω as the energy dissipation, in the case of interacting particle systems.

Remark: one of the anonymous referees pointed out that the scope of our investigation can be substantially extended, because: *“most quantum transport effects could be recast into a classical simulation picture of nonlocal collisions”*, cf. [34,35].

## 2. The Dissipation Function

Consider a system whose “microscopic” state is described by a dynamical system

(1) $$\dot{x}=V\left(x\right)\;;\;x\in M\subset R^{n}\;;\;\;M=\mathrm{phase}\mathrm{space}$$

Let (1) generate a flow on M, i.e., a map $S^{t}:M\to M$, such that $S^{t}\left(x\right),\;t\in R$ is the solution of (1) at time *t* with initial condition x. Further properties of M and V(x) will be specified when needed. Let $\mu_{0}$ be a probability measure absolutely continuous w.r.t. the Lebesgue measure dx, and denote by $f_{0}\left(x\right)$ its density function, i.e., $d\mu_{0}\left(x\right)=f_{0}\left(x\right)dx$. A measure μ is called invariant if $\mu \left(S^{-t}E\right)=\mu \left(E\right)$ for any measurable set E⊂M and for all t∈R. If $\mu_{0}$ is not invariant, it evolves under the dynamics, so that at time *t* it is expressed by $\mu_{t}\left(E\right)=\mu_{0}\left(S^{-t}E\right)$. Provided the flow is sufficiently smooth, $\mu_{t}$ is absolutely continuous, and has density $f_{t}\left(x\right)$ that satisfies [27]:

(2) $$\frac{\partial f_{t}}{\partial t}\left(x\right)=f_{t}\left(x\right)\;\Omega^{f_{t}}\left(x\right),$$

where $\Omega^{f_{t}}\left(x\right)$ is the dissipation function, defined by:

(3) $$\Omega^{f_{t}}\left(x\right):=-\left[\Lambda \left(x\right)+V\left(x\right)\cdot \nabla \ln f_{t}\left(x\right)\right]\;.$$

and

(4) Λ(x):=divV(x),

denotes the phase space variation rate. Its negative, −Λ(x), is called phase space contraction rate. In the case the dynamical systems represents an equilibrium system, with equilibrium distribution $\mu_{0}$, so that $f_{t}\left(x\right)\equiv f_{0}\left(x\right)$, the dissipation function identically vanishes, $\Omega^{f_{0}}\left(x\right)\equiv 0$. It is obviously true also the converse: if $\Omega^{f_{0}}\left(x\right)\equiv 0$ then $f_{0}\left(x\right)$ is an invariant density. In the general case, the solution of (2), is given by [27]:

(5) $$f_{t}\left(x\right)=\exp\left\{\Omega_{-t,0}^{f_{0}}\left(x\right)\right\}f_{0}\left(x\right),$$

where, for every phase function O we use the notation

(6) $$O_{r,s}\left(x\right)=\int_{r}^{s}O\left(S^{\tau }x\right)d\tau \;\;\mathrm{hence}\Omega_{-t,0}^{f_{0}}\left(x\right)=\int_{-t}^{0}\Omega^{f_{0}}\left(S^{\tau }x\right)d\tau .$$

Given any observable O:M→R, we define its ensemble average according to the measure $d\mu_{t}\left(x\right)=f_{t}\left(x\right)dx$ as

(7) $${\langle O\rangle }_{t}=\int_{M}O\left(x\right)f_{t}\left(x\right)dx\;.$$

Under suitable conditions, discussed in Section 6, this average can be computed using the dissipation function as follows [27]:

(8) $${\langle O\rangle }_{t}={\langle O\rangle }_{0}+\int_{0}^{t}{\langle \left(O\circ S^{\tau }\right)\cdot \Omega^{f_{0}}\rangle }_{0}d\tau \;.$$

Equation (8) gives the response of an equilibrium system, characterized by the distribution $\mu_{0}$, invariant for the dynamics $\dot{x}=V_{0}\left(x\right)$, which at time 0 is perturbed so that its dynamics becomes Equation (1). The measure $\mu_{0}$ is not invariant under the new dynamics, hence it evolves together with the averages representing the observables. At time *t* the initial measure has turned into $\mu_{t}$, and the initial average ${\langle O\rangle }_{0}$ into ${\langle O\rangle }_{t}$.

In the long time limit, the response may be given by a non equilibrium invariant distribution. As (8) is exact, the existence of such an asymptotic average is equivalent, as a necessary and sufficient condition called ΩT-mixing, to the convergence of the following limit:

(9) $$\lim_{t\to \infty }\int_{0}^{t}{\langle \left(O\circ S^{\tau }\right)\cdot \Omega^{f_{0}}\rangle }_{0}d\tau =L_{O}\in R\;.$$

for any observable O. A sufficient condition for the existence of $L_{O}$ is [26]:

(10) $${\langle \left(O\circ S^{t}\right)\;\Omega^{f_{0}}\rangle }_{0}=o\left(t^{-1}\right),$$

A further useful asymptotic property is obtained by considering two observables O and Q. In this case, we say that the system is *T*-mixing [26] if

(11) $$\lim_{t\to \infty }\left[{\langle (O\circ S^{t})Q\rangle }_{0}-{\langle (O\circ S^{t})\rangle }_{0}{\langle Q\rangle }_{0}\right]=0.$$

We observe that setting $Q=\Omega^{f_{0}}$ in (11) we obtain a weak form of ΩT-mixing, namely

(12) $$\lim_{t\to \infty }{\langle \left(O\circ S^{t}\right)\;\Omega^{f_{0}}\rangle }_{0}=0,$$

given that one has, cf. Section 6 and [27]:

(13) $${\langle \Omega^{f_{0}}\rangle }_{0}=0\;.$$

Obviously, *T*-mixing implies weak ΩT-mixing. We observe that these properties may hold on a specific set of observables for a given $f_{0}$ but not for other initial distributions.

A fundamental difference between *T*-mixing and the mixing property of standard ergodic theory is that *T*-mixing concerns a the decay of correlations w.r.t. the initial state $\mu_{0}$, hence it may properly describe the irreversible relaxation of a given system to a stationary state. Mixing, instead, corresponds to a decay of correlations within a given steady state, since it refers to an invariant measure [5]. We refer to Refs. [5,27] for a broader discussion of these issues.

### 2.1. Examples

The physical meaning of the Dissipation Function can be appreciated by considering some special choices of vector field V, and of initial probability density $f_{0}$. First, consider the NEMD systems for which Ω has been introduced, to see that in such cases it is directly related to the instantaneous dissipative flux. The simplest of these systems have the following equations of motion:

(14) $$\begin{array}{ccc} {\dot{q}}_{i} & = & p_{i}/m+C_{i}\left(\Gamma \right)\cdot F_{e}\;,\;i=1,...,N\\ {\dot{p}}_{i} & = & F_{i}+D_{i}\left(\Gamma \right)\cdot F_{e}-\alpha p_{i} \end{array}$$

where Γ=(q,p) collectively represents all coordinates **q** and momenta **p** of the *N* particles, and $F_{e}$ is the external driving field, coupled to the particles via the “charges’’ $C_{i}\left(\Gamma \right)$ and $D_{i}\left(\Gamma \right)$. The term α is a Lagrange multiplier meant to implement some constraint, so that the system reaches a steady state despite the driving field. In the NEMD literature α is said to implement a deterministic, time reversible thermostat [6]. If this thermostat is set to 0, one obtains the adiabatic equations of motion:

(15) $$\begin{array}{ccc} {\dot{q}}_{i} & = & p_{i}/m+C_{i}\left(\Gamma \right)\cdot F_{e}\;,\;i=1,...,N\\ {\dot{p}}_{i} & = & F_{i}+D_{i}\left(\Gamma \right)\cdot F_{e} \end{array}$$

which imply a continuous growth of energy. Then, one must assume that the support of $f_{0}$ is densely explored by a single equilibrium trajectory [30].

Denoting by Φ the internal interaction potential energy of the *N* particles, the dissipative flux, J, is obtained from the time derivative of the internal energy

$$H_{0}=\sum_{i=1}^{N}\frac{p_{i}\cdot p_{i}}{2m}+\Phi \left(q\right)$$

under the dynamics (15), which represents how much energy has to be dissipated in order to keep the steady state. The result is:

(16) $${\dot{H}}_{0}^{ad}=\sum_{i=1}^{N}\left(\frac{p_{i}}{m}\cdot D_{i}-F_{i}\cdot C_{i}\right)\cdot F_{e}\equiv -JV\cdot F_{e}$$

where *V* is the volume of the system.

Consider a few common examples.

#### 2.1.1. Microcanonical Ensemble

For the isoenergetic dynamics, in which the internal energy is conserved, i.e., $H_{0}$ is fixed and ${\dot{H}}_{0}=0$, the equilibrium ensemble $f_{0}$ is microcanonical. Therefore, for an equilibrium Hamiltonian *N* particle system in *d* dimensions, one has [30]:

(17) $$\alpha \left(\Gamma \right)=-\frac{J\left(\Gamma \right)V\cdot F_{e}}{2K(\Gamma )}$$

where *K* is the kinetic energy of the *N* particles, while

(18) $$\Lambda \left(\Gamma \right)=\sum_{i=1}^{N}\left(\frac{\partial C_{i}\left(\Gamma \right)}{\partial q_{i}}+\frac{\partial D_{i}\left(\Gamma \right)}{\partial p_{i}}\right)\cdot F_{e}-dN\alpha \left(\Gamma \right)+O\left(\frac{1}{N}\right)=-dN\alpha \left(\Gamma \right)+O\left(\frac{1}{N}\right)$$

as in the original paper [36]. Then, one may write:

$$\Omega_{t,t+\tau }^{\left(0\right)}\left(\Gamma \right)=\ln\frac{f_{0}\left(S^{t}\Gamma \right)}{f_{0}\left(S^{t+\tau }\Gamma \right)}-\Lambda_{t,t+\tau }\left(\Gamma \right)=-\Lambda_{t,t+\tau }\left(\Gamma \right)$$

As even the O(1/N) correction in Equation (18) is proportional to α, one observes that in this case both Ω and Λ are proportional to the rate of energy dissipation (17).

#### 2.1.2. Gaussian Isokinetic System

In this case the kinetic energy *K* is fixed, and one may formally write $K=\left(dN-d-1\right)k_{B}T=\left(dN-d-1\right)/\beta$, if also the center of mass is fixed, to prevent contributions from drift, so that:

(19) $$\alpha \left(\Gamma \right)=-\frac{({\dot{H}}_{0}\left(\Gamma \right)+J\left(\Gamma \right)V\cdot F_{e})\beta }{dN-d-1}$$

In turn, Λ is still expressed by (18), with a different O(1/N) term. In this case, $f_{0}$ writes:

$$f_{0}\left(\Gamma \right)=\frac{1}{Q}e^{-\beta H_{0}}\delta \left(K\left(\Gamma \right)-K_{0}\right)$$

where *Q* is the normalization constant. The result for the dissipation function is:

(20) $${\bar{\Omega }}_{0,\tau }\left(\Gamma \right)=\frac{\beta }{\tau }\int_{0}^{\tau }{\dot{H}}_{0}\left(S^{s}\Gamma \right)ds-{\bar{\Lambda }}_{0,\tau }\left(\Gamma \right)=-{\bar{\left(J\cdot F_{e}\right)}}_{0,\tau }V\beta +O\left(\frac{1}{N}\right)$$

where the bar over the observables denotes time average. Combining this with Equations (18) and (19), one obtains

(21) $$\sigma \left(\Gamma \right)=-J\left(\Gamma \right)\cdot F_{e}V\beta +O\left(\frac{1}{N}\right)\;.$$

where σ is the entropy production, if local thermodynamic equilibrium is verified. We observe that −Λ and Ω differ by a term proportional to the fluctuating $\dot{H}$. If these fluctuations average to zero, the averages of −Λ and Ω coincide, however, depending on how the respective fluctuations are distributed, various relations may apply to one and not to the other. For instance, the steady state fluctuation relation may hold for Ω but not for Λ, [37,38].

#### 2.1.3. Nosé-Hoover Thermostat

In this case, there is one extra equation to be added to the equations of motion (14):

(22) $$\dot{\zeta }=\frac{1}{\tau }\left(2K\left(\Gamma \right)-dNk_{B}T\right)$$

where τ sets the relaxation time scale of the thermostat, and *T* is the imposed average temperature. Then, we may write:

(23) $$\zeta \left(\Gamma \right)=-\left({\dot{H}}_{0}\left(\Gamma \right)+\frac{J\left(\Gamma \right)V\cdot F_{e}}{2K(\Gamma )}\right)$$

and for unperturbed Hamiltonian dynamics, one may write:

(24) $$\Lambda \left(\Gamma \right)=\nabla \cdot \dot{\Gamma }+\frac{\partial \dot{\zeta }}{\partial \zeta }=-dN\zeta \;.$$

In this case, $f_{0}$ is given by

$$f_{0}\left(\Gamma \right)=\frac{1}{M}e^{-\beta (H_{0}+\frac{1}{2}Q\zeta^{2})}$$

where *M* is the normalization. It follows that:

$$\frac{f_{0}\left(\Gamma \right)}{f_{0}\left(S^{\tau }\Gamma \right)}=e^{\beta \int_{0}^{\tau }\left({\dot{H}}_{0}\left(S^{s}\Gamma \right)+Q\zeta \left(S^{s}\Gamma \right)\dot{\zeta }\left(S^{s}\Gamma \right)\right)ds}$$

which implies:

(25) $$\Omega \left(\Gamma \right)=-J\left(\Gamma \right)\cdot F_{e}V\beta \;.$$

We conclude that for equilibrium Hamiltonian dynamics, the driven nonequilibrium evolution is characterized by the dissipation function, which equals the entropy production, in case local thermodynamic equilibrium holds. Additionally, in this case −Λ and Ω differ by a term proportional to ${\dot{H}}_{0}$, and the same remarks concluding the previous subsection equally apply.

#### 2.1.4. Canonical Ensemble for Generic Dynamics

The canonical ensemble describes the equilibrium state of a Hamiltonian particle systems in contact with a heat bath at temperature $k_{B}T=\beta^{-1}$, with which it can exchange energy but not matter. Here, we consider a general case in which the vector field V(x) is not necessarily Hamiltonian, but there still is an energy function *H*. In addition, we assume that the initial distribution has the same form of the canonical Gibbs density:

(26) $$f_{0}\left(x\right)=\frac{e^{-\beta H(x)}}{Z_{\beta }},$$

$Z_{\beta }$ being the normalization constant. Then, the Dissipation Function reads:

(27) $$\Omega^{f_{0}}\left(x\right)=\beta \dot{H}\left(x\right)-\Lambda \left(x\right),$$

where we use the notation $\dot{H}\left(x\right)=V\left(x\right)\cdot \nabla H\left(x\right)$. The Dissipation Function $\Omega^{f_{0}}$ is not necessarily zero, in this case, and the density evolves as follows, cf. (5):

$$f_{t}\left(x\right)=f_{0}\left(x\right)\exp\left\{\beta \left(H\left(x\right)-H\left(S^{-t}x\right)\right)-\Lambda_{-t,0}\left(x\right)\right\}=\frac{\exp\left\{-\beta H(S^{-t}x)\right\}}{Z_{t}\left(x\right)}.$$

where: $Z_{t}^{-1}\left(x\right)=\exp\left\{-\Lambda_{-t,0}\left(x\right)\right\}Z_{\beta }^{-1}$. In the case of Hamiltonian dynamics, $x=\left(q,p\right)\in R^{2n}$, V(x)=J∇H(x) where J is a symplectic matrix, we have $\dot{H}\left(x\right)=V\left(x\right)\cdot \nabla H\left(x\right)\equiv 0$ and Λ(x)≡0. Thus, in the equilibrium canonical ensemble, where (26) is invariant and the phase space volume is conserved, the dissipation function is identically zero $\Omega^{f_{0}}\left(x\right)\equiv 0$.

**Figure 1:**
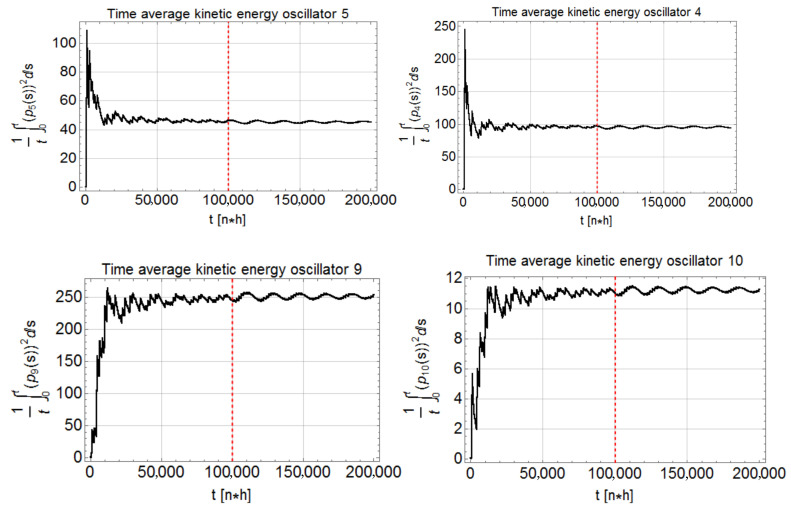
System of linear oscillators, α=1, subjected to a Nosé–Hoover thermostat, (28), with N=30, m=1, $\omega_{0}^{2}=2$, τ=2 and T=100. Time average of kinetic energy of oscillators i=4,5,9,10 versus time. The dependence of the asymptotic values on *i* demonstrate the violation of equipartition. The dashed vertical line marks the instant of time at which the constant $\omega_{0}^{2}$ is changed to $\omega_{0}^{2}+\delta \omega_{0}^{2}$ to check whether the oscillator “temperature’’ also changes. It appears that it does not.

The extended canonical equilibrium distribution of the Nosé–Hoover thermostatted system can be framed in this way: its dynamics includes a degree of freedom ζ that represents the heat bath, and system plus bath constitute a canonical equilibrium state. For instance, consider a system made of *N* identical noninteracting one-dimensional oscillators in contact with a bath at temperature *T*, implemented by a single Nosé–Hoover thermostat. This thermostat introduces an indirect coupling among the oscillators, which however does not represent a proper interaction leading to LTE. We illustrate these facts considering both linear and nonlinear oscillators, i.e., potential energies $U\left(q\right)=-\omega_{0}^{2}q^{\alpha +1}/\left(\alpha +1\right)$, with α=1 and α=3. The equations of motion are

(28) $$\left\{\begin{array}{c} {\dot{q}}_{i}=\frac{p_{i}}{m},\\ {\dot{p}}_{i}=-m\;\omega_{0}^{2}\;q_{i}^{\alpha }-\zeta p_{i},\;i=1,\ldots ,N,\\ \dot{\zeta }=\frac{1}{\tau }\left(\frac{2K(p)}{T}-1\right), \end{array}\right.$$

where *T* is the target kinetic energy per particle,

$$K\left(p\right)=\frac{1}{2mN}\sum_{i=1}^{N}p_{i}^{2}$$

is the kinetic energy per particle, and τ the relaxation time. In order to elucidate the non-thermodynamic nature of the model (28), we simulated a system of N=30 oscillators in the linear case, α=1, with m=1, $\omega_{0}^{2}=2$, τ=2 and T=100. As expected because of the improper particles coupling expressed by ζ, we observe no equipartition; cf. Figure 1, which shows that the time average ${\bar{K}}_{i}$ of the kinetic energies of single particles substantially change with their label *i*, while the time average $\bar{K}$ of the total is equal to *T* within numerical errors. The same happens also with α=3.

**Figure 2:**
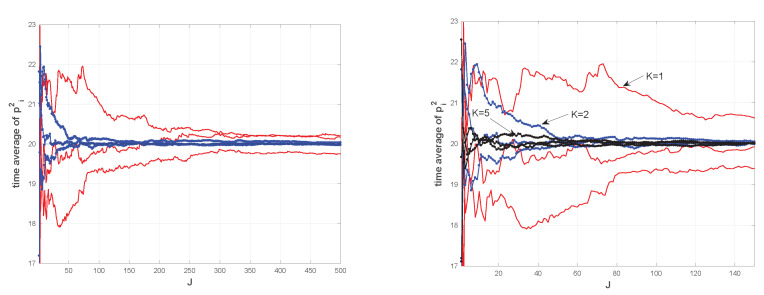
System with N=10 coupled oscillators, with α=3, m=1, $\omega_{0}^{2}=1$. The temperature forced by the Nosé–Hoover thermostat is T=20, and the response time τ=1. The plots represent the time averages $\frac{1}{J}\sum_{j=1}^{J}p_{i}^{2}\left(t_{j}\right)$, of the kinetic energies of three particles (i=1,4,8) sampled at $t_{j}=10^{3}\cdot j$, for increasing *J*. In the left panel, where J=500, the outer continuous (red online) lines correspond to the coupling parameter k=1, while the inner dotted lines (blue online) correspond to k=2. In the right panel the focus is on a shorter interval, J=150, where the relaxation phase can be better appreciated. In this case the outer lines (red online) correspond to k=1, the middle lines (blue online) correspond to k=2, the inner line (black) correspond to k=5. Larger coupling enhances the convergence rate towards equipartition, as expected.

To promote LTE in this system, we introduce nearest neighbour interactions as:

(29) $${\dot{p}}_{i}=-m\;\omega_{0}^{2}\;q_{i}^{\alpha }+k\left(q_{i-1}-2q_{i}+q_{i+1}\right)-\zeta p_{i},\;i=1,\ldots ,N,$$

in place of the second of (28), taking k>0 as the coupling strength, and $q_{0}=q_{n}$, $q_{n+1}=q_{0}$ as boundary conditions. Unlike the previous non-interacting model, the linear (α=1) and nonlinear (α=3) cases behave in a different way. While still absent in the linear case, equipartition is established with the cubic local field. Figure 2 shows the time averages $\frac{1}{J}\sum_{j=1}^{J}p_{i}^{2}\left(t_{j}\right)$ of the kinetic energies of some particles sampled form a long trajectory of the coupled system with N=10. We consider the cases with k=1,2,5 in order to make evident the role of the coupling. These simulations show that increasing the particles coupling *k* speeds up the equipartition process. Changing the oscillators frequency, instead, has no effect on equipartition, cf. Figure 1.

Now, focusing on the energy $H_{0}=\frac{1}{2m}\sum_{i=1}^{N}p_{i}^{2}+\frac{m}{2}\omega_{0}^{2}\sum_{i=1}^{N}q_{i}^{2}$ of the system of oscillators alone, i.e., without the bath, and taking $f_{0}$ as

(30) $$f_{0}\left(\Gamma \right)=\frac{1}{Z_{0}}e^{-\beta H_{0}\left(\Gamma \right)}$$

in order to define a kind of reduced dissipation function for the dynamics with the bath, one obtains:

$${\hat{\Omega }}_{0}^{f_{0}}=\zeta \left(N-\frac{\beta }{m}\sum_{i=1}^{N}p_{i}^{2}\right)=N\zeta \left(1-2T^{-1}K\left(p\right)\right)=-\Theta^{2}N\zeta \dot{\zeta }=-N\Theta^{2}\frac{d}{dt}\left(\frac{\zeta^{2}}{2}\right),$$

which is not identically zero even in the equilibrium state. There is no immediate physical interpretation of the quantity ${\hat{\Omega }}_{0}^{f_{0}}$, but it clearly shows that, in this framework, the thermostat has to be included to properly speak of equilibrium.

#### 2.1.5. Hamiltonian and Gradient Vector Field with Canonical and Gaussian $f_{0}$

In the case of Hamiltonian dynamics perturbed by a gradient term [39]:

(31) V(x)=J∇H(x)−∇L(x),

where the unperturbed equilibrium density should be chosen consistently with the constraints that remain in place when the perturbation is switched on, in order to implement the above-mentioned ergodic consistency condition. For instance, if the system temperature remains fixed, one may take the canonical distribution, $f_{0}\left(x\right)=e^{-\beta H(x)}/Z$, that implies:

$$\Omega^{f_{0}}\left(x\right)=\Delta L\left(x\right)-\beta \nabla L\left(x\right)\cdot \nabla H\left(x\right)\;,$$

ΔL=−Λ being the Laplacian of *L*. One observes that $\Omega^{f_{0}}$ only depends on *L* if ∇L is orthogonal to ∇H. If ∇L is in addition a Hamiltonian perturbation, the perturbed steady state remains equal to the unperturbed equilibrium. Now, taking an initial uncorrelated Gaussian distribution:

(32) $$f_{0}\left(x\right)=\prod_{i=1}^{n}\frac{1}{\sqrt{2\pi }\sigma_{i}}e^{-\frac{{\left(x_{i}-\mu_{i}\right)}^{2}}{2\sigma_{i}^{2}}}$$

one obtains:

(33) $$\Omega^{f_{0}}=-\Lambda \left(x\right)+\sum_{i=1}^{n}V_{i}\left(x\right)\frac{x_{i}-\mu_{i}}{\sigma_{i}^{2}}=\Delta L\left(x\right)+\sum_{i=1}^{n}\left(\sum_{j=i}^{N}J_{ij}\frac{\partial H(x)}{\partial x_{j}}-\frac{\partial L(x)}{\partial x_{i}}\right)\frac{x_{i}-\mu_{i}}{\sigma_{i}^{2}},$$

$J_{ij}$ being the entries of J. Again, a Hamiltonian perturbation such that

(34) $$\sum_{j=i}^{N}J_{ij}\frac{\partial H(x)}{\partial x_{j}}-\frac{\partial L(x)}{\partial x_{i}}=0\;,\;\forall i=1,...,N$$

implies that the Gaussian (32) is invariant under the dynamics. These two examples constitute elementary warnings against too quick thermodynamic interpretations of generic particle systems, much as it may be interesting in certain circumstances [40].

### 2.2. The Dissipation Function and the Phase Space Contraction Rate

The Dissipation Function satisfies the Non Equilibrium Partition identity [27]:

(35) $${\langle \exp\left(-\Omega_{-t,0}^{f_{0}}\right)\rangle }_{0}=1$$

Using Jensen inequality, we can also write:

(36) $$\exp\left[-{\langle \Omega_{-t,0}^{f_{0}}\rangle }_{0}\right]\leq 1$$

obtaining:

$${\langle \Omega_{-t,0}^{f_{0}}\rangle }_{0}\geq 0$$

in accordance with both our physical intuition on the dissipation of energy in macroscopic systems and with the interpretation of this quantity as a Kullback–Leibler divergence between the initial and the evolved probability distribution in phase space [27].

One motivation to derive relations like these, see e.g., Refs. [27,30] where various others are derived, resides in the fact that they are only based on the dynamics and on the initial probability distribution, whatever phenomenon they represent, hence they hold very generally. At the same time, this also means that blind thermodynamic interpretations are to be avoided, although no difficulty arises from a purely dynamical systems standpoint.

The distinction between the dissipation function and the phase space contraction rate −Λ becomes particularly evident when considering particle systems. In fact, the steady state phase space average of −Λ equals the steady state entropy production of certain NEMD models [6,19], and it can be expressed as:

(37) $$e_{p}=-\int_{M}\Lambda \left(x\right)d\mu_{(}x),$$

μ being the corresponding invariant measure, and $e_{p}$ the steady state entropy production or the energy dissipation. On these grounds, Gallavotti and Cohen proposed −Λ as the steady state definition of entropy production tout court, [41], followed by Ruelle [23]. Earlier, a similar idea had been contemplated by Andrey [20], who referred to the variation in time of the Gibbs entropy. The Gibbs entropy, however, is not directly related, in general, to the entropy of a nonequilbrium system [42,43,44]. (Note: this statement does not concern the formally analogous definition of entropy common in the theory of stochastic processes, since they are not defined in phase space but in the space of observables.) Therefore, −Λ cannot be directly related, in general, to the entropy production, when considering time dependent states or steady state fluctuations, cf. Section 2.1 and Refs. [27,30,38].

On the other hand, it is interesting to investigate both −Λ and Ω as properties of dynamical systems, in a kind of extension of the thermodynamic formalism [33], that refers the non-equilibrium language.

Given an initial absolutely continuous probability measure $d\mu_{0}\left(x\right)=f_{0}\left(x\right)dx$ on the phase space M, and letting $\mu_{t}$ be the evolution of $\mu_{0}$ at time *t*, the corresponding average of the dissipation function is:

(38) $${\langle \Omega^{f_{0}}\rangle }_{t}=\int_{M}\Omega^{f_{0}}d\mu_{t}=-{\langle \Lambda \rangle }_{t}-{\langle V\cdot \nabla \ln f_{0}\rangle }_{t}$$

which in the case of a uniform density $f_{0}$ yields:

(39) $${\langle \Omega^{f_{0}}\rangle }_{t}=-{\langle \Lambda \rangle }_{t}$$

In this case, even $\Omega^{f_{0}}$ does not need to represent any entropy production rate: the equilibrium dynamics would have at least to be phase space volumes preserving, for $\Omega^{f_{0}}$ to be related to the dissipation of energy [30]. Using Equation (8) and the fact that ${\langle \Omega^{f_{0}}\rangle }_{0}=0$, we may also write:

(40) $$-{\langle \Lambda \rangle }_{t}=-\int_{0}^{t}{\langle \Omega^{f_{0}}\left(\Lambda \circ S^{\tau }\right)\rangle }_{0}d\tau \;$$

In the case of a generic probability density $f_{0}$, the exact response formula (8) and (40) yield:

(41) $${\langle \left(-\Lambda -\Omega^{f_{0}}\right)\circ S^{t}\rangle }_{0}=-{\langle \Lambda \rangle }_{t}-{\langle \Omega^{f_{0}}\rangle }_{t}={\langle V\cdot \nabla \ln f_{0}\rangle }_{0}+\int_{0}^{t}{\langle \Omega^{f_{0}}\left[\left(V\cdot \nabla \ln f_{0}\right)\circ S^{\tau }\right]\rangle }_{0}d\tau \;$$

which quantifies the difference between the mean phase space contraction rate and the energy dissipation in time. In the case ΩT-mixing, Equation (9), holds, *t* can be replaced by *∞* in (41), obtaining the asymptotic difference between the two quantities:

(42) $$\lim_{t\to \infty }\left[{\langle -\Lambda \rangle }_{t}-{\langle \Omega^{f_{0}}\rangle }_{t}\right]={\langle V\cdot \nabla \ln f_{0}\rangle }_{0}+\int_{0}^{\infty }{\langle \Omega^{f_{0}}\left[\left(V\cdot \nabla \ln f_{0}\right)\circ S^{\tau }\right]\rangle }_{0}d\tau \;.$$

From the definition of dissipation function $\Omega^{f_{0}}$, i.e., Equation (3) with t=0, one also observes that

(43) $$\left(-\Lambda -\Omega^{f_{0}}\right)\circ S^{s}={\left.\frac{d}{dt}\ln f_{0}\circ S^{t}\right|}_{t=s}$$

Therefore, the time integrated difference of −Λ and $\Omega^{f_{0}}$ merely amounts to a boundary term:

(44) $$\int_{0}^{t}\left(-\Lambda -\Omega^{f_{0}}\right)\left(S^{s}x\right)ds=\ln f_{0}\left(x\right)-\ln f_{0}\left(S^{t}x\right)$$

which, however, may bear noticeably affect the statistic of fluctuations: the distribution of such boundary terms may, for instance, imply the validity of the FR for $\Omega^{f_{0}}$ and not for −Λ, cf. [37,38,45].

## 3. Exact Response and GK Formalism

In this section, we compare the classical linear response theory, or Green–Kubo formalism, with the exact response represented by Equation (8). Consider a perturbed vector field

(45) $$G_{\epsilon }\left(x\right)=G_{0}\left(x\right)+\epsilon \;G_{p}\left(x\right),$$

where ϵ may or may not be small. Then, the Dissipation Function can be written as:

(46) $$\Omega_{G_{\epsilon }}^{f_{0}}\left(x\right)=\Omega_{G_{0}}^{f_{0}}\left(x\right)+\epsilon \;\Omega_{G_{p}}^{f_{0}}\left(x\right).$$

and the evolution of a probability density $f_{0}\left(x\right)$ is given by

(47) $$\begin{array}{cc} f_{t}\left(x;\epsilon \right) & =f_{0}\left(x\right)\exp\left[\int_{-t}^{0}d\tau \;\Omega_{G_{0}}^{f_{0}}\left(S_{\epsilon }^{\tau }x\right)\right]\exp\left[\epsilon \int_{-t}^{0}d\tau \;\Omega_{G_{p}}^{f_{0}}\left(S_{\epsilon }^{\tau }x\right)\right]. \end{array}$$

where $S_{\epsilon }^{t}$ denotes the flow of the vector field $G_{\epsilon }\left(x\right)$. Thus, the exact response Equation (8) reads

(48) $$\begin{array}{cc} {\langle O\rangle }_{t;\epsilon } & :=\int O\left(x\right)f_{t}\left(x;\epsilon \right)dx\\ & ={\langle O\rangle }_{0}+\int_{0}^{t}{\langle \left(O\circ S_{\epsilon }^{\tau }x\right)\cdot \Omega_{G_{0}}^{f_{0}}\left(x\right)\rangle }_{0}d\tau +\epsilon \;\int_{0}^{t}{\langle \left(O\circ S_{\epsilon }^{\tau }x\right)\cdot \Omega_{G_{p}}^{f_{0}}\left(x\right)\rangle }_{0}d\tau . \end{array}$$

In case $G_{0}$ derives from a Hamiltonian $H_{0}$, the unperturbed dynamics preserves phase space volumes, and one can write:

(49) $$\Omega_{G_{0}}^{f_{0}}=-G_{0}\cdot \nabla \ln f_{0}=-\frac{1}{f_{0}}\left[f_{0},H_{0}\right]=\frac{1}{f_{0}}L_{0}f_{0}$$

where $L_{0}=\left[H_{0},\cdot \right]$ is the Liouvillean corresponding to $H_{0}$ and [·,·] denotes the associated Poisson brackets. Then, we may define the perturbation operator $L_{p}$ as:

(50) $$\Omega_{G_{p}}^{f_{0}}=-G_{p}\cdot \nabla \ln f_{0}=-\frac{1}{f_{0}}\left[f_{0},H_{p}\right]=\frac{1}{f_{0}}L_{p}f_{0}$$

so that the (total) dissipation function can be written as:

(51) $$\Omega_{G_{\epsilon }}^{f_{0}}=\frac{1}{f_{0}}\left(L_{0}+\epsilon L_{p}\right)f_{0}\;,$$

Substituting (51) in (48) yields:

(52) $$\begin{array}{ccc} {\langle O\rangle }_{t;\epsilon } & = & {\langle O\rangle }_{0}+\int_{0}^{t}{\langle (O\left(S_{\epsilon }^{\tau }x\right)\frac{1}{f_{0}\left(x\right)}L_{0}f_{0}\left(x\right)\rangle }_{0}d\tau +\epsilon \;\int_{0}^{t}{\langle (O\left(S_{\epsilon }^{\tau }x\right)\frac{1}{f_{0}\left(x\right)}L_{p}f_{0}\left(x\right)\rangle }_{0}d\tau \;\\ & = & {\langle O\rangle }_{0}+\int_{0}^{t}d\tau \;\int_{M}dx\;O\left(S_{\epsilon }^{\tau }x\right)L_{0}f_{0}\left(x\right)+\epsilon \;\int_{0}^{t}d\tau \;\int_{M}dx\;O\left(S_{\epsilon }^{\tau }x\right)L_{p}f_{0}\left(x\right) \end{array}$$

and in case the initial distribution $f_{0}\left(x\right)$ is the equilibrium state of the unperturbed system, so that $L_{0}f_{0}\equiv 0$, one obtains:

(53) $${\langle O\rangle }_{t;\epsilon }={\langle O\rangle }_{0}+\epsilon \;\int_{0}^{t}d\tau \int_{M}dx\;O\left(S_{\epsilon }^{\tau }x\right)\;L_{p}f_{0}\left(x\right)\;.$$

Provided the response Equation (8) applies, the formulae (52) and (53) for the evolution of phase space averages are both exact: there is no assumption on the size of ϵ and no approximation has been made. An approximate formula can be obtained in the small-ϵ limit, starting from Equation (47), by truncating to first order the exponential expansion, and recalling that $\Omega_{G_{0}}^{f_{0}}\left(x\right)\equiv 0$. This yields:

(54) $${\tilde{f}}_{t}\left(x;\epsilon \right):=f_{0}\left(x\right)+\epsilon f_{0}\left(x\right)\int_{-t}^{0}d\tau \;\Omega_{G_{p}}^{f_{0}}\left(S_{\epsilon }^{\tau }x\right)=f_{0}\left(x\right)+\epsilon f_{0}\left(x\right)\int_{0}^{t}d\tau \;\Omega_{G_{p}}^{f_{0}}\left(S_{\epsilon }^{-\tau }x\right)\;.$$

where the tilde symbol stresses that this is but an approximation of the probability density at time *t*, which is not guaranteed to be a probability density itself. Nevertheless, multiplying (54) by O and integrating over the phase space, one gets:

(55) $${\langle O\rangle }_{t;\epsilon }^{∼}:=\int_{M}O\left(x\right){\tilde{f}}_{t}\left(x;\epsilon \right)dx={\langle O\rangle }_{0}+\epsilon \int_{0}^{t}d\tau \;{\langle O\left(x\right)\Omega_{G_{p}}^{f_{0}}\left(S_{\epsilon }^{-\tau }x\right)\rangle }_{0}$$

In order to keep the truncation error bounded in time, one may assume that the time integral exists. For instance, ${\langle O\left(x\right)\Omega_{G_{p}}^{f_{0}}\left(S_{\epsilon }^{-t}x\right)\rangle }_{0}$ maybe of order $o(t^{-1})$, which is reminiscent of the ΩT-mixing condition. The difference between the exact response formula, (48), and the approximate one, (55), amounts to:

(56) $${\langle O\rangle }_{t;\epsilon }-{\langle O\rangle }_{t;\epsilon }^{∼}=\epsilon \int_{0}^{t}\;d\tau \left\{{\langle O\left(S_{\epsilon }^{\tau }x\right)\cdot \Omega_{G_{p}}^{f_{0}}\left(x\right)\rangle }_{0}-{\langle O\left(x\right)\Omega_{G_{p}}^{f_{0}}\left(S_{\epsilon }^{-\tau }x\right)\rangle }_{0}\right\}\;,$$

that, in general, does not vanish, and may grow large in time.

May the difference of responses Equation (56) become negligible, in some limit? Applying the transformation $S_{\epsilon }^{-\tau }x=y$ to the second integrand of (56), one obtains:

(57) $${\langle O\left(x\right)\Omega_{G_{p}}^{f_{0}}\left(S_{\epsilon }^{-\tau }x\right)\rangle }_{0}=\int_{M}O\left(x\right)\Omega_{G_{p}}^{f_{0}}\left(S_{\epsilon }^{-\tau }x\right)f_{0}\left(x\right)dx=\int_{M}O\left(S_{\epsilon }^{\tau }y\right)\Omega_{G_{p}}^{f_{0}}\left(y\right)f_{0}\left(S_{\epsilon }^{\tau }y\right)\left|J_{S_{\epsilon }^{\tau }}\left(y\right)\right|dy\;,$$

where $\left|J_{S_{\epsilon }^{\tau }}\left(y\right)\right|=1$ since we are dealing with Hamiltonian dynamics–being $\left|J_{S_{\epsilon }^{\tau }}\left(y\right)\right|$ the Jacobian determinant. Thus, Equation (56) can be rewritten as:

(58) $${\langle O\rangle }_{t;\epsilon }-{\langle O\rangle }_{t;\epsilon }^{∼}=\epsilon \int_{0}^{t}\;d\tau \int_{M}O\left(S_{\epsilon }^{\tau }x\right)\Omega_{G_{p}}^{f_{0}}\left(x\right)\left[f_{0}\left(x\right)-f_{0}\left(S_{\epsilon }^{\tau }x\right)\right]dx$$

For every finite *t*, this quantity vanishes in the ϵ→0 limit but, unlike the case of the Green–Kubo theory of linear response, the flow used in (58) is the perturbed one, $S_{\epsilon }^{\tau }$, not the unperturbed $S_{0}^{\tau }$.

As a matter of fact, the success of the Green–Kubo theory is based on its ability to compute the response by averaging with respect to the equilibrium ensemble for the unperturbed dynamics, and by simulating such an unperturbed dynamics [46]. The procedure, illustrated in many textbooks, including [46,47], uses the canonical ensemble for the unperturbed Hamiltonian $H_{0}$, $Z_{0}^{-1}\exp\left\{-\beta H_{0}\right\}$, and assumes that the parameter ϵ is the magnitude of and external force $F^{e}$. Within the framework of NEMD and Equation (14), one starts from the unthermostatted, α=0 situation. Without loss of generality, the external driving $F_{e}$ can be written as a scalar quantity, since its direction can be embedded in the coefficients $C_{i}$ and $D_{i}$. Then, the unthermostatted perturbed equations are derived from a Hamiltonian, $H=H_{0}+H_{p}$. Writing x=(q,p) to distinguish configuration from momenta, $G_{p}$ is obtained deriving $H_{p}$:

(59) $$C_{i}F_{e}=\frac{\partial H_{p}}{\partial p_{i}}\;;\;D_{i}F_{e}=-\frac{\partial H_{p}}{\partial q_{i}}$$

and the following holds:

(60) $$0=\Lambda =\frac{\partial \dot{q}}{\partial q}+\frac{\partial \dot{p}}{\partial p}=\sum_{i=1}^{N}\left\{\frac{\partial C_{i}}{\partial q_{i}}+\frac{\partial D_{i}}{\partial p_{i}}\right\}F_{e}\;,\;\mathrm{hence}\;\;\sum_{i=1}^{N}\left\{\frac{\partial C_{i}}{\partial q_{i}}+\frac{\partial D_{i}}{\partial p_{i}}\right\}=0\;.$$

In this case, one has:

(61) $$\Omega_{G_{p}}^{f_{0}}=-G_{p}\cdot \nabla \ln f_{0}=\frac{1}{f_{0}}\frac{\partial f_{0}}{\partial H_{0}}G_{p}\cdot \nabla H_{0}=-\beta \sum_{i=1}^{N}\left[C_{i}\dot{\frac{\partial H_{0}}{\partial q_{i}}}+D_{i}\dot{\frac{\partial H_{0}}{\partial p_{i}}}\right]F_{e}=-\beta VJF_{e}$$

which equals Equation (16), and shows again that $\Omega_{G_{\epsilon }}^{f_{0}}$, not −Λ, represents that energy dissipation rate. Now, following the Green–Kubo approach [46,47], which rests on the fact that

(62) $$\left[H_{0},H_{p}\right]=\left[H,H_{p}\right]\;,$$

one may use the unperturbed dynamics, and eventually write:

(63) $$\begin{array}{c} {\langle O\rangle }_{t;F_{e}}^{∼}={\langle O\rangle }_{0}-\beta F_{e}\int_{0}^{t}d\tau \;{\langle \left(O\circ S_{0}^{t-\tau }\right)\sum_{i=1}^{N}\left[C_{i}\dot{\frac{\partial H_{0}}{\partial q_{i}}}+D_{i}\dot{\frac{\partial H_{0}}{\partial p_{i}}}\right]\rangle }_{0} \end{array}$$

(64) $$\begin{array}{c} \;={\langle O\rangle }_{0}-\beta VF_{e}\int_{0}^{t}d\tau \;{\langle J\left(O\circ S_{0}^{t-\tau }\right)\rangle }_{0} \end{array}$$

to first order in $\epsilon =F_{e}$, for a generic observable O. The function $R\left(t\right)={\langle J\left(O\circ S_{0}^{t}\right)\rangle }_{0}$ is called the response function. In particular, the difference in (56) between exact and linear response can be expressed as:

(65) $${\langle O\rangle }_{t;\epsilon }-{\langle O\rangle }_{t;\epsilon }^{∼}=\epsilon \int_{0}^{t}\;d\tau {\langle \Omega_{G_{p}}^{f_{0}}\left[O\circ S_{\epsilon }^{\tau }-O\circ S_{0}^{t-\tau }\right]\rangle }_{0}$$

That the full dynamics $S_{\epsilon }^{\tau }$ can be replaced by the equilibrium dynamics $S_{0}^{\tau }$ is a far from trivial fact. In fact, van Kampen raised one of the best known objections to this theory, based on the observation that particles dynamics are typically chaotic [48]. He maintained that while there is no doubt that linear response works in practice, the derivations a la Green and Kubo do not explain why. He stated that: *“Linear response theory does provide expressions for the phenomenological coefficients, but I assert that it arrives at these expressions by a mathematical exercise, rather than by describing the actual mechanism which is responsible for the response”* [48]. It fact, to justify the Green–Kubo calculation, one must invoke non trivial ingredients, such as the large value of *N*, which makes the perturbation per degree of freedom negligible, and the mingling of contrasting contributions operated by chaos, see e.g., [45,49,50]. As such conditions actually hold in macroscopic particles systems under quite variegated conditions, the Green–Kubo reasoning explains the success of linear response.

### 3.1. Exact Response and GK Formalism: Example 1

Consider a particle in a two-dimensional space, whose unperturbed Hamiltonian is $H_{0}=\left(p_{x}^{2}+p_{y}^{2}\right)/2$. Take the canonical ensemble $f_{0}=Z_{0}^{-1}\exp\left(-\beta H_{0}\right)$ as the initial distribution. Introduce an external field with Hamiltonian $H_{p}=-Eq_{x}$, as a perturbation to obtain $H=H_{0}+H_{p}$. The corresponding equations of motion are:

(66) $$\left\{\begin{array}{c} {\dot{q}}_{x}=p_{x},\\ {\dot{q}}_{y}=p_{y},\\ {\dot{p}}_{x}=E,\\ {\dot{p}}_{y}=0, \end{array}\right.$$

with the dissipation function

$$\Omega_{E}^{f_{0}}=\Omega_{0}^{f_{0}}+\Omega_{p}^{f_{0}}=\beta Ep_{x},$$

in which the momentum in *x*-direction plays the role of internal dissipative flux with *E* the corresponding thermodynamic force, i.e., $J=-p_{x}$ and $F_{e}=E$. Note that the present example is an instance of the adiabatic system (15), in which $C_{1}=C_{2}=0$ and $D_{1}=1$, $D_{2}=0$, see (59). Sticking to the notation of Section 2.1 in the following we denote by $\Gamma =(q_{x},q_{y},p_{x},p_{y})$ the phase space points and observe that the solution of (66) with initial condition Γ gives $p_{x}\left(\Gamma ,t\right)=p_{x}+Et$ and $p_{y}\left(\Gamma ,t\right)=p_{y}$.

Then, thanks to Equation (5), we compute the time evolution of the probability distribution function $f_{t}\left(\Gamma \right)$ starting from the integrated dissipation function:

$$\Omega_{-t,0}^{f_{0}}\left(\Gamma \right)=\beta E\int_{-t}^{0}p_{x}\left(\Gamma ,s\right)ds=\beta Ep_{x}t-\beta E^{2}\frac{t^{2}}{2},$$

obtaining

$$f_{t}\left(\Gamma \right)=f_{0}\left(\Gamma \right)e^{\Omega_{-t,0}^{f_{0}}\left(\Gamma \right)}=f_{0}\left(\Gamma \right)\exp\left[\beta Ep_{x}t-\beta E^{2}\frac{t^{2}}{2}\right].$$

Following the discussion in Section 3, we expand the exponential in the limit of small external field *E*, and we neglect the terms of order higher than linear in *E*, to obtain the linear response for the evolving density:

$${\tilde{f}}_{t}\left(\Gamma \right)=f_{0}\left(\Gamma \right)\left(1+\beta Ep_{x}t\right).$$

The difference between exact and linear response can be further clarified by looking at the Liouville equation for $f_{t}\left(\Gamma \right)$ and proceeding a la Green–Kubo. Let us start introducing the Liouville operator of the perturbed system:

$$L=L_{0}+\Delta L,$$

where $L_{0}=p_{x}\partial_{q_{x}}+p_{y}\partial_{q_{y}}$ corresponds to the unperturbed system and $\Delta L\equiv L_{p}=E\partial_{p_{x}}$ corresponds to the perturbation. Splitting the evolving ensemble distribution function as:

$$f_{t}\left(\Gamma \right)=f_{0}\left(\Gamma \right)+\Delta f_{t}\left(\Gamma \right)$$

the Liouville equation takes the form:

$$\partial_{t}\left(f_{0}\left(\Gamma \right)+\Delta f_{t}\left(\Gamma \right)\right)+\left(L_{0}+\Delta L\right)\left(f_{0}\left(\Gamma \right)+\Delta f_{t}\left(\Gamma \right)\right)=0$$

Recalling that the equilibrium distribution $f_{0}\left(\Gamma \right)$ is invariant under the unperturbed dynamics—so that $\left(\partial_{t}+L_{0}\right)f_{0}\left(\Gamma \right)=0$—we obtain the evolution equation for the deviation $\Delta f_{t}\left(\Gamma \right)$:

$$\left(\partial_{t}+L_{0}\right)\Delta f_{t}\left(\Gamma \right)=-\Delta Lf_{0}\left(\Gamma \right)-\Delta L\Delta f_{t}\left(\Gamma \right).$$

Then, assuming that $\Delta L\Delta f_{t}(\Gamma )$ is negligible w.r.t. the other terms, we arrive at:

(67) $$\left(\partial_{t}+L_{0}\right)\Delta f_{t}\left(\Gamma \right)=-\Delta Lf_{0}\left(\Gamma \right).$$

Since $\Delta Lf_{0}\left(\Gamma \right)=E\partial_{p_{x}}\left[{Z_{0}}^{-1}\;\exp\left(-\beta H_{0}\right)\right]=-\beta Ep_{x}f_{0}\left(\Gamma \right)$, the Equation (67) can be rewritten as

$$\left(\partial_{t}+p_{x}\partial_{q_{x}}+p_{y}\partial_{q_{y}}\right)\Delta f_{t}\left(\Gamma \right)=\beta Ep_{x}f_{0}\left(\Gamma \right)$$

where $\beta f_{0}=-\partial f_{0}/\partial H_{0}$, $E=F_{e}$ and $p_{x}=-J$, according to standard notation. The solution to the evolution equation for the variation of the ensemble distribution function, is given by:

(68) $$\begin{array}{cc} \Delta f_{t}\left(\Gamma \right) & =-\int_{0}^{t}d\tau \exp\left[-\left(t-\tau \right)L_{0}\right]\Delta L\left(\tau \right)f_{0}\left(\Gamma \right)=-\beta E\int_{0}^{t}d\tau \exp\left[-\left(t-\tau \right)L_{0}\right]\left(-p_{x}\right)f_{0}\left(\Gamma \right)\\ & =-\beta F_{e}\int_{0}^{t}d\tau \exp\left[-\left(t-\tau \right)L_{0}\right]J\left(\Gamma \right)f_{0}\left(\Gamma \right), \end{array}$$

where we have used the fact that the external perturbation is not time dependent.

Let us now compute the phase space average of an observable O, using $f_{t}\left(\Gamma \right)=f_{0}\left(\Gamma \right)+\Delta f_{t}\left(\Gamma \right)$. We can write:

(69) $$\begin{array}{cc} {\langle O\rangle }_{t}^{∼} & ={\langle O\rangle }_{0}-\beta F_{e}\int_{0}^{t}d\tau \int_{M}d\Gamma f_{0}\left(\Gamma \right)J\left(\Gamma \right)\exp\left[-\left(t-\tau \right)L_{0}\right]O\left(\Gamma \right)\\ & ={\langle O\rangle }_{0}+\beta E\int_{0}^{t}d\tau \int_{M}d\Gamma f_{0}\left(\Gamma \right)p_{x}\left(O\circ S_{0}^{t-\tau }\left(\Gamma \right)\right)={\langle O\rangle }_{0}+\int_{0}^{t}d\tau {\langle \left(O\circ S_{0}^{t-\tau }\right)\cdot \Omega_{E}^{f_{0}}\rangle }_{0} \end{array}$$

noting that $S_{0}^{t}$ is the flow of the unperturbed dynamics. As an example, take $O=p_{x}$, and compute its time evolving linear response. Since ${\langle p_{x}\rangle }_{0}=0$ and ${\langle p_{x}^{2}\rangle }_{0}=\beta^{-1}$, we have:

(70) $$\begin{array}{c} {\langle p_{x}\rangle }_{t}^{∼}=\frac{\beta E}{Z_{0}}\int_{0}^{t}ds\int dp_{y}e^{-\frac{\beta }{2}p_{y}^{2}}\int dp_{x}e^{-\frac{\beta }{2}p_{x}^{2}}p_{x}^{2}=\beta E{\langle p_{x}^{2}\rangle }_{0}t=\beta E\frac{1}{\beta }t=Et \end{array}$$

Now, denoting by $S_{E}^{t}$ the flow of the perturbed system, and recalling that $p_{x}\circ S_{E}^{t}=p_{x}+Et$ and $\Omega_{E}^{f_{0}}=\beta Ep_{x}$, the exact response formula (8) yields:

(71) $$\begin{array}{cc} {\langle p_{x}\rangle }_{t} & =\int_{0}^{t}d\tau \int_{M}d\Gamma f_{0}\left(\Gamma \right)\left(p_{x}\circ S_{E}^{\tau }\left(\Gamma \right)\right)\Omega_{E}^{f_{0}}\left(\Gamma \right)=\beta E\int_{0}^{t}d\tau \int_{M}d\Gamma f_{0}\left(\Gamma \right)\left(p_{x}^{2}+Ep_{x}\tau \right)=\beta E{\langle p_{x}^{2}\rangle }_{0}t=Et \end{array}$$

For such an observable, linear and exact response coincide. In general, we have:

(72) $${\langle O\rangle }_{t}-{\langle O\rangle }_{t}^{∼}=\int_{0}^{t}ds{\langle \Omega_{E}^{f_{0}}\;\left[\left(O\circ S_{E}^{\tau }\right)-\left(O\circ S_{0}^{t-\tau }\right)\right]\rangle }_{0}.$$

For an analytic observable that do not depend on $q_{x}$ and $q_{y}$, we have:

(73) $$O\left(S_{E}^{t}\Gamma \right)=O\left(p_{x}+Et,p_{y}\right)=O\left(p_{x},p_{y}\right)+\sum_{k=1}^{+\infty }\frac{O^{\left(k\right)}\left(p_{x},p_{y}\right)}{k!}{\left(Et\right)}^{k},$$

assuming that $O=O(p_{x},p_{y})$ is an analytic function, and denoting by $O^{\left(k\right)}$ the k-th partial derivative of O with respect to $p_{x}$, at fixed $p_{y}$. The difference between the exact and the linear response would read:

(74) $${\langle O\rangle }_{t}-{\langle O\rangle }_{t}^{∼}=\int_{0}^{t}d\tau {\langle \Omega_{E}^{f_{0}}\left(\Gamma \right)\sum_{k=1}^{+\infty }\frac{O^{\left(k\right)}\left(p_{x},p_{y}\right)}{k!}{\left(E\tau \right)}^{k}\rangle }_{0}$$

Depending on the particular functional form of O this quantity may grow rapidly or less rapidly with *t* and, as long as $O(S_{E}^{t}\Gamma )$ exists at all *t*, this difference at fixed *t* goes to 0 as E→0.

### 3.2. Exact Response and GK Formalism: Example 2

Let the unperturbed Hamiltonian correspond to a harmonic oscillator $H_{0}=\left(p^{2}+\omega^{2}q^{2}\right)/2$, and let the perturbation take the form $H_{p}=-Eq$. The equations of motion of $H=H_{0}+H_{p}$ are:

(75) $$\left\{\begin{array}{c} \dot{q}=p,\\ \dot{p}=E-\omega^{2}q, \end{array}\right.$$

with flow

(76) $$S_{E}^{t}\left(\begin{array}{c} q\\ p \end{array}\right)=\left(\begin{array}{c} \left(q-\frac{E}{\omega^{2}}\right)\cos\omega \;t+\frac{p}{\omega }\sin\omega \;t+\frac{E}{\omega^{2}}\\ -\omega \left(q-\frac{E}{\omega^{2}}\right)\sin\omega \;t+p\cos\omega \;t \end{array}\right).$$

The dissipation function with initial distribution $f_{0}=e^{-\beta H_{0}}/Z_{0}$ is given by:

(77) $$\Omega_{E}^{f_{0}}=-G\cdot \nabla \ln f_{0}=\left(p,E-\omega^{2}q\right)\cdot \beta \left(\begin{array}{c} \omega^{2}q\\ p \end{array}\right)=\beta Ep$$

since the vector field is Hamiltonian and Λ vanishes. Moreover, we see that $\Omega_{E}^{f_{0}}$ coincides with the dissipation function $\Omega_{p}^{f_{0}}$ of the vector filed of $H_{p}$, because $f_{0}$ is the equilibrium distribution of $H_{0}$. Thus the exact and the linear response formulas are respectively given by:

(78) $${\langle O\rangle }_{t}={\langle O\rangle }_{0}+\int_{0}^{t}ds\;{\langle \beta Ep\;\left(O\circ S_{E}^{s}\right)\rangle }_{0}$$

and

(79) $${\langle O\rangle }_{t}^{∼}={\langle O\rangle }_{0}+\int_{0}^{t}ds\;{\langle \beta Ep\;\left(O\circ S_{0}^{t-s}\right)\rangle }_{0}\;.$$

Consider now O=q. In this case $O\circ S_{E}^{s}$ and $O\circ S_{0}^{s}$ are given by (76). For E≠0 we have:

(80) $$\begin{array}{cc} {\langle \beta Ep\;\left(O\circ S_{E}^{s}\right)\rangle }_{0} & =\int \int dq\;dp\frac{e^{-\beta (\frac{p^{2}}{2}+\frac{\omega^{2}}{2}q^{2})}}{Z_{0}}\beta Ep\;\left[\left(q-\frac{E}{\omega^{2}}\right)\cos\omega \;t+\frac{p}{\omega }\sin\omega \;t+\frac{E}{\omega^{2}}\right]\\ & =\frac{\beta E}{\omega }\sin\omega \;t\int_{-\infty }^{+\infty }dq\int_{-\infty }^{+\infty }dp\frac{e^{-\beta (\frac{p^{2}}{2}+\frac{\omega^{2}}{2}q^{2})}}{Z_{0}}p^{2}=\frac{E}{\omega }\sin\omega \;t, \end{array}$$

since ${\langle p\rangle }_{0}=\int \int dq\;dp\;f_{0}\left(q,p\right)p=0$, ${\langle q\rangle }_{0}=\int \int dq\;dp\;f_{0}\left(q,p\right)q=0$ and ${\langle p^{2}\rangle }_{0}=\int \int dq\;dp\;f_{0}\left(q,p\right)p^{2}=\beta^{-1}$. The response formula (78) gives

(81) $${\langle p\rangle }_{t}={\langle p\rangle }_{0}+\int_{0}^{t}ds\;\frac{E}{\omega }\sin\omega \;s=\frac{E}{\omega^{2}}\left(1-\cos\omega t\right).$$

Analogously, the unperturbed dynamics, given by E=0 in (76), yields:

(82) $$\begin{array}{cc} {\langle \beta Ep\;\left(O\circ S_{0}^{t-s}\right)\rangle }_{0} & =\int \int dq\;dp\frac{e^{-\beta (\frac{p^{2}}{2}+\frac{\omega^{2}}{2}q^{2})}}{Z_{0}}\beta Ep\;\left[q\cos\omega \;\left(t-s\right)+\frac{p}{\omega }\sin\omega \;\left(t-s\right)\right]=\frac{E}{\omega }\;\sin\omega \;\left(t-s\right), \end{array}$$

and from (79)

(83) $${\langle p\rangle }_{t}^{∼}={\langle p\rangle }_{0}+\int_{0}^{t}ds\;\frac{E}{\omega }\;\sin\omega \;\left(t-s\right)=\frac{E}{\omega^{2}}\left(1-\cos\omega t\right).$$

This shows that the perturbed and unperturbed responses of the observable O=p coincide. If we consider the observable O=qp, the situation is different. In the first place,

(84) $$\begin{array}{cc} O\circ S_{E}^{t} & =-\omega {\left(q-\frac{E}{\omega^{2}}\right)}^{2}\frac{1}{2}\sin2\omega t+p\left(q-\frac{E}{\omega^{2}}\right)\cos^{2}\omega t-p\left(q-\frac{E}{\omega^{2}}\right)\sin^{2}\omega t+p^{2}\frac{1}{2\omega }\sin2\omega t\\ \; & \;-\frac{E}{\omega }\left(q-\frac{E}{\omega^{2}}\right)\sin\omega t+p\frac{E}{\omega^{2}}\cos\omega t\;, \end{array}$$

that must be multiplied by *p* before taking the ensemble averages in (78) and (79). This gives a linear combination of monomials $p,\;pq,\;pq^{2},p^{2}q,\;p^{3}$, whose average with respect to ${\langle \cdot \rangle }_{0}$ vanishes, and a non vanishing term

$$p^{2}\frac{E}{\omega^{2}}\left[-\cos^{2}\omega t+\sin^{2}\omega t+\cos\omega t\right]$$

Thus, recalling that ${\langle p^{2}\rangle }_{0}=\beta^{-1}$, we have:

(85) $${\langle qp\rangle }_{t}=\beta E\int_{0}^{t}ds\frac{E}{\omega^{2}}{\langle p^{2}\rangle }_{0}\left[-\cos^{2}\omega s+\sin^{2}\omega s+\cos\omega s\right]=\frac{E^{2}}{\omega^{3}}\left[\sin\omega t+\frac{1}{2}\sin2\omega t\right].$$

On the other hand, from (84) with E=0, we obtain that $p(O\circ S_{0}^{t})$ is a linear combination of $q^{2}p,\;qp^{2},p^{3}$. This implies that ${\langle \beta Ep\;\left(O\circ S_{0}^{t-s}\right)\rangle }_{0}=0$ and, from (79), that ${\langle qp\rangle }_{t}^{∼}=0$, showing that the perturbed and unperturbed responses substantially differ in the case of O=qp, unless *E* is particularly small.

## 4. Formal Thermodynamics

Following Qian and collaborators [29], we now obtain various new relations for the dissipation function. To this end, suppose that the system admits an energy function H:M→R, $H\in C^{1}\left(R^{N},R\right)$. Let us introduce the set $\Gamma_{h}$ in phase space, defined as follows:

$$\Gamma_{h}=\left\{x\in R^{N}:H\left(x\right)\leq h\right\}$$

and its boundary:

$$\partial \Gamma_{h}=\left\{x\in R^{N}:H\left(x\right)=h\right\}$$

The set $\Gamma_{h}$ includes all points in phase space whose energy is equal to or lower than the threshold *h*, and $\partial \Gamma_{h}$ is its boundary, constituted by the phase points whose energy equals the threshold value. Introduce the measure of such sets w.r.t. the time evolving probability measure:

$$\mu_{t}\left(h\right)=\int_{\Gamma_{h}}f_{t}\left(x\right)dx$$

where $f_{t}\left(x\right)$ is a sufficiently well behaved probability density. Then, the function

(86) $$\frac{f_{t}\left(x\right)}{\mu_{t}\left(h\right)}\chi_{h}\left(x\right)\;;\;\chi_{h}=\left\{\begin{array}{cc} 1 & \mathrm{if}x\in \Gamma_{h}\\ 0 & \mathrm{if}x\notin \Gamma_{h} \end{array}\right.$$

where $\chi_{h}$ is the characteristic function of $\Gamma_{h}$, plays the role of a probability density in $\Gamma_{h}$. Using Reynolds Transport Theorem plus the regularity of the surface $\partial \Gamma_{h}$, one finds:

(87) $$\frac{d}{dh}\mu_{t}\left(h\right)=\mu_{t}^{\prime }\left(h\right)=\int_{\partial \Gamma_{h}}f_{t}\left(x\right)\frac{d\sigma }{∥\nabla H∥},$$

where dσ is the surface element area. This quantity can be interpreted as the measure w.r.t. the density $f_{t}\left(x\right)$ of the constant energy surface $\partial \Gamma_{h}$. The time derivative along a trajectory of the energy function H(x) is given by:

$$\dot{H}\left(x\right)=V\left(x\right)\cdot \nabla H\left(x\right)$$

hence we may define a formal heat flux through the boundary $\partial \Gamma_{h}$ as:

$${\dot{Q}}_{t}\left(h\right)=-\frac{1}{\mu_{t}^{\prime }\left(h\right)}\int_{\partial \Gamma_{h}}V\left(x\right)\cdot \nabla H\left(x\right)f_{t}\left(x\right)\frac{d\sigma }{∥\nabla H∥}$$

If in addition, we introduce the formal temperature as the inverse of the logarithmic derivative of the constant energy volume w.r.t. the energy parameter *h*:

$$T_{t}\left(h\right)={\left(\frac{d}{dh}\ln\mu_{t}\left(h\right)\right)}^{-1}=\frac{\mu_{t}\left(h\right)}{\mu_{t}^{\prime }\left(h\right)}$$

we can relate the ensuing formal entropy production rate to the average of the dissipation function concerning the set $\Gamma_{h}$:

(88) $$e_{p}\left(h;t\right)=\frac{{\dot{Q}}_{t}\left(h\right)}{T_{t}\left(h\right)}=\frac{1}{\mu_{t}\left(h\right)}\int_{\Gamma_{h}}\Omega^{f_{t}}\left(x\right)f_{t}\left(x\right)dx=\frac{1}{\mu_{t}\left(h\right)}{\langle \Omega^{f_{t}}\cdot \chi_{h}\rangle }_{t}$$

This result is readily obtained as follows:

$${\dot{Q}}_{t}\left(h\right)=-\frac{1}{\mu_{t}^{\prime }\left(h\right)}\int_{\partial \Gamma_{h}}V\left(x\right)\cdot \nabla H\left(x\right)f_{t}\left(x\right)\frac{d\sigma }{∥\nabla H∥}=-\frac{1}{\mu_{t}^{\prime }\left(h\right)}\int_{\Gamma_{h}}\nabla \cdot \left(f_{t}\left(x\right)V\left(x\right)\right)dx$$

where the equality is guaranteed by Divergence Theorem, ∇H/∥∇H∥ being the unit vector everywhere normal to the surface $\partial \Gamma_{h}$, and by the identity $\nabla \cdot \left(f_{t}\left(x\right)V\left(x\right)\right)=-\Omega^{f_{t}}\left(x\right)f_{t}\left(x\right)$. Then Equations (3) and (5), yield:

(89) $$\begin{array}{cc} \Omega^{f_{t}}\left(x\right) & =-\left(\Lambda \left(x\right)+V\left(x\right)\cdot \nabla \ln f_{t}\left(x\right)\right)=-\left[\Lambda \left(x\right)+V\left(x\right)\cdot \nabla \ln(f_{0}\left(x\right)\exp\left\{\Omega_{-t,0}^{f_{0}}\left(x\right)\right\}\right]=\\ & =-\left[\Lambda \left(x\right)+V\left(x\right)\cdot \nabla (\ln f_{0}\left(x\right)+\Omega_{-t,0}^{f_{0}}\left(x\right))\right] \end{array}$$

that leads to:

(90) $$\Omega^{f_{t}}\left(x\right)=\Omega^{f_{0}}\left(x\right)-V\left(x\right)\cdot \nabla \Omega_{-t,0}^{f_{0}}\left(x\right).$$

Therefore, we can now write:

(91) $$\begin{array}{cc} e_{p}\left(h;t\right) & =\frac{1}{\mu_{t}\left(h\right)}\int_{M}\Omega^{f_{t}}\left(x\right)\chi_{h}\left(x\right)f_{t}\left(x\right)dx=\frac{1}{\mu_{t}\left(h\right)}\int_{M}\left(\Omega^{f_{0}}\left(x\right)-V\left(x\right)\cdot \nabla \Omega_{-t,0}^{f_{0}}\left(x\right)\right)\chi_{h}\left(x\right)f_{t}\left(x\right)dx\\ & =\frac{1}{\mu_{t}\left(h\right)}\left(\int_{M}\Omega^{f_{0}}\left(x\right)\chi_{h}\left(x\right)f_{t}\left(x\right)dx-\int_{M}V\left(x\right)\cdot \nabla \Omega_{-t,0}^{f_{0}}\left(x\right)\chi_{h}\left(x\right)f_{t}\left(x\right)dx\right) \end{array}$$

The first addend within the brackets can be reworked using the response formula (8), where $\Omega^{f_{0}}$ restricted to $\Gamma_{h}$ is considered like any other phase function:

(92) $$\int_{M}\Omega^{f_{0}}\left(x\right)\chi_{h}\left(x\right)f_{t}\left(x\right)dx=\int_{M}\Omega^{f_{0}}\left(x\right)\chi_{h}\left(x\right)f_{0}\left(x\right)dx+\int_{0}^{t}\int_{M}\Omega^{f_{0}}\left(S^{\tau }x\right)\chi_{h}\left(S^{\tau }x\right)\Omega^{f_{0}}\left(x\right)f_{0}\left(x\right)dxd\tau$$

while for the second contribution, we have:

(93) $$\begin{array}{cc} \int_{M}V\left(x\right)\cdot \nabla \Omega_{-t,0}^{f_{0}}\left(x\right)\chi_{h}\left(x\right)f_{t}\left(x\right)dx & =\int_{M}V\left(x\right)\cdot \nabla \Omega_{-t,0}^{f_{0}}\left(x\right)\chi_{h}\left(x\right)f_{0}\left(x\right)e^{\Omega_{-t,0}^{f_{0}}\left(x\right)}dx\\ & =\int_{M}V\left(x\right)\cdot \nabla e^{\Omega_{-t,0}^{f_{0}}\left(x\right)}\chi_{h}\left(x\right)f_{0}\left(x\right)dx \end{array}$$

Rearranging the two contributions, the first integral in Equation (91) becomes:

(94) $$\begin{array}{c} {\langle \Omega^{f_{t}}\chi_{h}\rangle }_{t}={\langle \Omega^{f_{0}}\chi_{h}\rangle }_{0}+\int_{0}^{t}d\tau \int_{M}dx\;\Omega^{f_{0}}\left(S^{\tau }x\right)\chi_{h}\left(S^{\tau }x\right)\Omega^{f_{0}}\left(x\right)f_{0}\left(x\right)-{\langle V\cdot \nabla e^{\Omega_{-t,0}^{f_{0}}}\chi_{h}\rangle }_{0} \end{array}$$

and finally:

(95) $$\begin{array}{cc} e_{p}\left(h;t\right) & =\frac{1}{\mu_{t}\left(h\right)}\left\{{\langle \left(\Omega^{f_{0}}-V\cdot \nabla e^{\Omega_{-t,0}^{f_{0}}}\right)\chi_{h}\rangle }_{0}+\int_{0}^{t}{\langle \Omega^{f_{0}}\left(\Omega^{f_{0}}\chi_{h}\right)\circ S^{\tau }\rangle }_{0}d\tau \right\}\;. \end{array}$$

Letting *h* grow in Equation (88), these conditional averages converge to the average on M, yielding:

$$\lim_{h\to \infty }e_{p}\left(h;t\right)={\langle \Omega^{f_{t}}\rangle }_{t}=0$$

where (13) has been used. This is consistent with the fact that in this model no “energy” can flow out of M, or in M from outside.

Note that such a reasoning actually does not refer to a physical flow: in the first place, it occurs in phase space, rather than in real space; furthermore the dynamics does not need to describe any physical phenomenon. Therefore, the thermodynamic interpretation of the above relations should be taken with a grain of salt [43,44], while it may be suggestive in the analysis of dynamical systems, analogously to the thermodynamic formalism [33].

### The Case with Constant or Slowly-Varying Volume Contraction

Assume that the probability density $f_{0}$ is continuous with compact support M, and take $V\left(x\right)=V_{0}\left(x\right)+\epsilon V_{1}\left(x\right)$, with $V_{0}$, $V_{1}$ sufficiently regular, $\Lambda_{0}\left(x\right)\equiv \mathrm{div}V_{0}\left(x\right)=-\lambda_{0}<0$. Assume also that the system is ΩT-mixing. To compute ${\langle \Omega_{V}^{f_{0}}\rangle }_{t}$ up to O(ϵ), observe that:

(96) $${\langle \Omega_{V}^{f_{0}}\rangle }_{t}=\int_{0}^{t}{\langle \Omega_{V}^{f_{0}}\left(\Omega_{V}^{f_{0}}\circ S_{\epsilon }^{\tau }\right)\rangle }_{0}d\tau =\int_{0}^{t}{\langle \Omega_{V}^{f_{0}}\left(\Omega_{V_{0}}^{f_{0}}\circ S_{\epsilon }^{\tau }\right)\rangle }_{0}d\tau +\epsilon \int_{0}^{t}{\langle \Omega_{V}^{f_{0}}\left(\Omega_{V_{1}}^{f_{0}}\circ S_{\epsilon }^{\tau }\right)\rangle }_{0}d\tau$$

where

$$\Omega_{V}^{f_{0}}\left(x\right)=\Omega_{V_{0}}^{f_{0}}\left(x\right)+\epsilon \;\Omega_{V_{1}}^{f_{0}}\left(x\right)=-\lambda_{0}-V_{0}\left(x\right)\cdot \nabla \ln f_{0}\left(x\right)+\epsilon \;\Omega_{V_{1}}^{f_{0}}\left(x\right)$$

Substituting in (96), we have:

(97) $$\begin{array}{cc} {\langle \Omega_{V}^{f_{0}}\rangle }_{t} & =\lambda_{0}^{2}\;t\\ & +\lambda_{0}\int_{0}^{t}d\tau \int_{M}dxf_{0}\left(x\right)V_{0}\left(S_{\epsilon }^{\tau }x\right)\cdot \nabla \ln f_{0}\left(S_{\epsilon }^{\tau }x\right)+\lambda_{0}\int_{0}^{t}d\tau \int_{M}dxf_{0}\left(x\right)V_{0}\left(x\right)\cdot \nabla \ln f_{0}\left(x\right)\\ & +\int_{0}^{t}d\tau \int dxf_{0}\left(x\right)\left(V_{0}\left(S_{\epsilon }^{\tau }x\right)\cdot \nabla \ln f_{0}\left(S_{\epsilon }^{\tau }x\right)\right)\left(V_{0}\left(x\right)\cdot \nabla \ln f_{0}\left(x\right)\right)+O\left(\epsilon \right) \end{array}$$

The second integral can be computed explicitly taking $f_{0}\left(x\right)=0$ on ∂M:

(98) $${\langle V_{0}\cdot \nabla \ln f_{0}\rangle }_{0}=\int_{M}V_{0}\left(x\right)\cdot \nabla f_{0}\left(x\right)dx=\int_{M}\mathrm{div}\left(V_{0}\left(x\right)f_{0}\left(x\right)\right)dx-\int_{M}\mathrm{div}V_{0}\left(x\right)f_{0}\left(x\right)dx\;.$$

As the divergence of $V_{0}$ equals the constant $-\lambda_{0}$, and $\int_{M}f_{0}\left(x\right)dx=1$, we have:

(99) $${\langle V_{0}\cdot \nabla \ln f_{0}\rangle }_{0}=\lambda_{0}+\int_{M}\mathrm{div}\left(V_{0}\left(x\right)f_{0}\left(x\right)\right)dx.$$

The integral in the previous equation is, by the Divergence Theorem, equal to

$$\int_{\partial M}f_{0}\left(x\right)V_{0}\left(x\right)\cdot n(x)d\sigma =0,$$

Then

(100) $${\langle V_{0}\cdot \nabla \ln f_{0}\rangle }_{0}=\lambda_{0},$$

and

(101) $$\begin{array}{cc} {\langle \Omega_{V}^{f_{0}}\rangle }_{t} & =2\lambda_{0}^{2}\;t+\lambda_{0}\int_{0}^{t}{\langle V_{0}\left(S_{\epsilon }^{\tau }\left(x\right)\right)\cdot \nabla \ln f_{0}\left(S_{\epsilon }^{\tau }\left(x\right)\right)\rangle }_{0}d\tau +\\ & +\int_{0}^{t}{\langle \left(V_{0}\left(S_{\epsilon }^{\tau }\left(x\right)\right)\cdot \nabla \ln f_{0}\left(S_{\epsilon }^{\tau }\left(x\right)\right)\right)\left(V_{0}\left(x\right)\cdot \nabla \ln f_{0}\left(x\right)\right)\rangle }_{0}d\tau +O\left(\epsilon \right). \end{array}$$

Averaging Λ one has instead:

$${\langle \Lambda \rangle }_{t}=\int_{M}\Lambda \left(x\right)f_{t}\left(x\right)dx=\int_{M}\left(\lambda_{0}-\epsilon \;\mathrm{div}V_{1}\left(x\right)\right)f_{t}\left(x\right)dx=\lambda_{0}+O\left(\epsilon \right),$$

since to first order, our vector field has a constant phase space volumes contraction rate.

**Example** **1.** *The simplest case is obtained when the initial distribution is uniform, i.e., $f_{0}\left(x\right)=$ constant, as if the “equilibrium” vector field vanishes. In that case the integrals in (101) vanish, and*

$${\langle \Omega_{V}^{f_{0}}\rangle }_{t}=2\lambda_{0}^{2}\;t+O\left(\epsilon \right)\;.$$

**Example** **2.** *Suppose the coordinates in the initial ensemble are independent, i.e.,*

$$f_{0}\left(x\right)=\prod_{i=1}^{n}g_{i}\left(x_{i}\right),$$

*with $g_{i}$ being one dimensional density functions. Then,*

(102) $$\frac{\partial }{\partial x_{k}}\ln f_{0}\left(x\right)=\frac{g_{k}^{\prime }\left(x_{k}\right)}{g_{k}\left(x_{k}\right)}$$

*and Equation (101) can be rewritten as follows:*

(103) $$\begin{array}{cc} {\langle \Omega_{V}^{f_{0}}\rangle }_{t} & =2\lambda_{0}^{2}\;t+\lambda_{0}\sum_{i=1}^{n}\int_{0}^{t}{\langle {V_{0}}_{i}\left(S_{\epsilon }^{\tau }x\right)\frac{g_{i}^{\prime }\left({\left(S_{\epsilon }^{\tau }x\right)}_{i}\right)}{g_{i}\left({\left(S_{\epsilon }^{\tau }x\right)}_{i}\right)}\rangle }_{0}d\tau +\\ & +\sum_{i,j=1}^{n}\int_{0}^{t}{\langle {V_{0}}_{i}\left(S_{\epsilon }^{\tau }x\right){V_{0}}_{j}\left(x\right)\frac{g_{i}^{\prime }\left({\left(S_{\epsilon }^{\tau }x\right)}_{i}\right)}{g_{i}\left({\left(S_{\epsilon }^{\tau }x\right)}_{i}\right)}\frac{g_{j}^{\prime }\left(x_{j}\right)}{g_{j}\left(x_{j}\right)}\rangle }_{0}d\tau +O\left(\epsilon \right). \end{array}$$

*In the special case of Gaussian marginals*

(104) $$g_{k}\left(x_{k}\right)=\frac{1}{\sqrt{2\pi }\sigma_{k}}e^{-\frac{{\left(x_{k}-\mu_{k}\right)}^{2}}{2\sigma_{k}^{2}}}\;;\;\frac{g_{i}^{\prime }\left(x_{i}\right)}{g_{i}\left(x_{i}\right)}=-\frac{\left(x_{k}-\mu_{k}\right)}{\sigma_{k}^{2}}$$

*we have:*

(105) $$\begin{array}{cc} {\langle \Omega_{V}^{f_{0}}\rangle }_{t} & =2\lambda_{0}^{2}\;t-\lambda_{0}\sum_{i=1}^{n}\sigma_{i}^{-2}\int_{0}^{t}{\langle {V_{0}}_{i}\left(S_{\epsilon }^{\tau }x\right)\left({\left(S_{\epsilon }^{\tau }x\right)}_{i}-\mu_{i}\right)\rangle }_{0}d\tau +\\ \; & +\sum_{i,j=1}^{n}{\left(\sigma_{i}\sigma_{j}\right)}^{-2}\int_{0}^{t}{\langle {V_{0}}_{i}\left(S_{\epsilon }^{\tau }x\right){V_{0}}_{j}\left(x\right)\left({\left(S_{\epsilon }^{\tau }x\right)}_{i}-\mu_{i}\right)\left(x_{j}-\mu_{j}\right)\rangle }_{0}d\tau +O\left(\epsilon \right). \end{array}$$

*The simplest example can be obtained with ϵ=0 and ${V_{0}}_{i}\left(x\right)=-\lambda_{i}x_{i}$, $\lambda_{i}>0$, and assuming $\mu_{k}=0$. Then, observing that the i-th component of $S_{0}^{\tau }x$, can be written as ${\left(S_{0}^{\tau }x\right)}_{i}=e^{-\lambda_{i}t}x_{i}$, Equation (105) becomes:*

(106) $$\begin{array}{cc} {\langle \Omega_{V}^{f_{0}}\rangle }_{t} & =2\lambda_{0}^{2}\;t+\lambda_{0}\sum_{i=1}^{n}\sigma_{i}^{-2}\lambda_{i}\int_{0}^{t}e^{-2\lambda_{i}\tau }{\langle x_{i}^{2}\rangle }_{0}d\tau +\\ \; & +\sum_{i,j=1}^{n}{\left(\sigma_{i}\sigma_{j}\right)}^{-2}\lambda_{i}\lambda_{j}\int_{0}^{t}e^{-2(\lambda_{i}+\lambda_{j})\tau }{\langle x_{i}^{2}x_{j}^{2}\rangle }_{0}d\tau , \end{array}$$

*where $\lambda_{0}=\sum_{i=1}^{n}\lambda_{i}$. In addition, recalling that ${\langle x_{i}^{2}\rangle }_{0}=\sigma_{i}^{2}$ and ${\langle x_{i}^{2}x_{j}^{2}\rangle }_{0}=\sigma_{i}^{2}\sigma_{j}^{2}$, we have:*

(107) $${\langle \Omega_{V}^{f_{0}}\rangle }_{t}=2\lambda_{0}^{2}\;t+\frac{1}{2}\lambda_{0}n-\frac{1}{2}\lambda_{0}\sum_{i=1}^{n}e^{-2\lambda_{i}t}+\sum_{i,j=1}^{n}\frac{\lambda_{i}\lambda_{j}}{\lambda_{i}+\lambda_{j}}-\sum_{i,j=1}^{n}\frac{\lambda_{i}\lambda_{j}}{\lambda_{i}+\lambda_{j}}e^{-2(\lambda_{i}+\lambda_{j})t}.$$

*The previous result could have been obtained directly from Equation (96), noting that:*

$$\Omega_{V_{0}}^{f_{0}}\left(x\right)=-\lambda_{0}-\sum_{i=1}^{n}\frac{\lambda_{i}}{\sigma_{i}^{2}}x_{i}^{2}.$$

*Equation (107) shows that the average dissipation function ${\langle \Omega_{V}^{f_{0}}\rangle }_{t}$ diverges as t→∞, because the dynamics has the global attractor x=0. Thus, $\Omega_{V}^{f_{0}}$ tends to the non vanishing phase space contraction rate $-\lambda_{0}$. That is not the case for ϵ>0.*

## 5. Simple Attractors and Small Dissipation

Consider the dynamical system (1) on a compact phase space M containing a set of attracting fixed points $A=\{x_{1},\ldots ,x_{k}\}$ with (open) basins of attraction $B_{j}=\left\{x\in M:S^{t}x\to x_{j},\;t\to +\infty \right\},j=1,\ldots ,k$, such that $\cup_{i=1}^{k}B_{j}=M$, modulo a set of zero Lebesgue measure. Let $f_{0}$ be the initial probability density and $f_{t}$ its evolution at time *t*. Recalling that the basins of attraction are pairwise disjoint, for any continuous phase function *C* we can write:

(108) $${\langle C\rangle }_{t}=\int_{M}C\left(x\right)f_{t}\left(x\right)dx=\int_{M}C\left(S^{t}x\right)f_{0}\left(x\right)dx=\sum_{i=1}^{k}\int_{B_{i}}C\left(S^{t}x\right)f_{0}\left(x\right)dx.$$

Taking the limit, by bounded convergence we have:

(109) $$\lim_{t\to \infty }{\langle C\rangle }_{t}=\sum_{i=1}^{k}w_{i}C\left(x_{i}\right)\;;\;\mathrm{where}\;\;\;\sum_{i=1}^{k}w_{i}=1\;,\;w_{i}\equiv \int_{B_{i}}f_{0}\left(x\right)dx>0$$

This shows that the asymptotic mean of *C* is the weighted mean of the values of *C* on the attracting fixed points, with weights given by the $\mu_{0}$-probability measure of the corresponding basins of attraction. In other words, the probability measure $d\mu_{t}\left(x\right)=f_{t}\left(x\right)\;dx$ converges weakly in time to $\nu =\sum_{i=1}^{k}w_{i}\delta_{x_{i}}$, with $\delta_{x_{i}}$ the atomic measure on $x_{i}$. In this case it is easy to compute the correlation function of *A* and *C* w.r.t. $\mu_{0}$:

(110) $${\langle (A\circ S^{t})C\rangle }_{0}-{\langle (A\circ S^{t})\rangle }_{0}{\langle C\rangle }_{0}$$

that is used to define T-mixing and, taking $C=\Omega^{f_{0}}$, to define ΩT-mixing, cf. Equations (10) and (11). The t→∞ limit of (110) then yields:

(111) $$\lim_{t\to \infty }\left[{\langle (A\circ S^{t})C\rangle }_{0}-{\langle (A\circ S^{t})\rangle }_{0}{\langle C\rangle }_{0}\right]=\sum_{i=1}^{k}A\left(x_{i}\right)\left[\int_{B_{i}}C\left(x\right)f_{0}\left(x\right)dx-w_{i}\int_{M}C\left(x\right)f_{0}\left(x\right)dx\right]$$

and, assuming that also $\Omega^{f_{0}}$ is continuous,

(112) $$\lim_{t\to \infty }\left[{\langle \left(C\circ S^{t}\right)\Omega^{f_{0}}\rangle }_{0}-{\langle (C\circ S^{t})\rangle }_{0}{\langle \Omega^{f_{0}}\rangle }_{0}\right]=\lim_{t\to \infty }{\langle \left(C\circ S^{t}\right)\Omega^{f_{0}}\rangle }_{0}=\sum_{i=1}^{k}C\left(x_{i}\right)\int_{B_{i}}\Omega^{f_{0}}\left(x\right)f_{0}\left(x\right)dx$$

since ${\langle \Omega^{f_{0}}\rangle }_{0}=0$.

**Remark** **1.** *For k>1, the response from a single initial condition is not unique and depends on the initial distribution $f_{0}$. Hence, T-mixing does not hold, since it requires the long time averages to be independent of the initial condition [26]. On the other hand, *Ω*T-mixing holds, since it is necessary and sufficient for the convergence of the full phase space averages to their asymptotic limit, i.e., (109).*

Now, consider a case with small dissipation:

(113) $$\dot{x}=V\left(x;\epsilon \right)=V_{0}\left(x\right)+\epsilon V_{1}\left(x\right),$$

where ϵ is small, and

(114) $$\mathrm{div}V_{0}\left(x\right)=0\;;\;V_{0}\left(x\right)\cdot \nabla f_{0}\left(x\right)=0$$

Then, the unperturbed dynamics $\dot{x}=V_{0}\left(x\right)$ preserves the volume and the initial density, since $\Omega_{V_{0}}^{f_{0}}\left(x\right)\equiv 0$, while the dissipation function only depends on the perturbation $\epsilon V_{1}$:

(115) $$\Omega_{V}^{f_{0}}\left(x\right)=\epsilon \;\Omega_{V_{1}}^{f_{0}}\left(x\right)=-\epsilon \left[\mathrm{div}V_{1}\left(x\right)+V_{1}\left(x\right)\cdot \nabla \ln f_{0}\left(x\right)\right]$$

Let the initial probability density $f_{0}$ be positive for every x in the phase space M, a condition at times called ergodic consistency [30]. In this case, the probability density at time *t* is given by:

$$f_{t}\left(x\right)=f_{0}\left(x\right)\exp\left[\epsilon \int_{-t}^{0}\Omega_{V_{1}}^{f_{0}}\left(S_{\epsilon }^{\tau }x\right)d\tau \right],$$

where $S_{\epsilon }^{\tau }$ is the flow of (113). Taking α∈(0,1) and $t_{\epsilon }=1/\epsilon^{\alpha }$, it can be shown that the difference between the evolved density and the initial one is $O(\epsilon^{1-\alpha })$, in the ϵ-dependent time interval bound by $-t_{\epsilon }$, provided $\Omega_{V}^{f_{0}}$ is continuous in M. In particular, one has:

(116) $$\left|\int_{-t_{\epsilon }}^{0}\Omega_{V_{1}}^{f_{0}}\left(S_{\epsilon }^{\tau }x\right)dt_{1}\right|\leq \max_{x\in M}\left|\Omega_{V_{1}}^{f_{0}}\left(x\right)\right|t_{\epsilon }={\tilde{\Omega }}_{V_{1}}^{f_{0}}t_{\epsilon }$$

which defines the maximum magnitude of $\Omega_{V_{1}}^{f_{0}}$ in M. Consequently, for $0<t\leq t_{\epsilon }$:

(117) $$|f_{t}\left(x\right)-f_{0}\left(x\right)|=f_{0}\left(x\right)\left|\exp\left(\epsilon \int_{-t}^{0}\Omega_{V_{1}}^{f_{0}}\left(S_{\epsilon }^{\tau }x\right)d\tau \right)-1\right|\leq f_{0}\left(x\right)\left[\exp\left({\tilde{\Omega }}_{V_{1}}^{f_{0}}\epsilon^{1-\alpha }\right)-1\right]=f_{0}\left(x\right)\;O\left(\epsilon^{1-\alpha }\right)$$

We can use this inequality to bound from above the variation of an observable of the system at hand. For a continuous function *B*, one obtains:

(118) $$\left|{\langle B\rangle }_{t}-{\langle B\rangle }_{0}\right|\leq \int_{M}\left|B\left(x\right)\right|\left(\exp\left[{\tilde{\Omega }}_{V_{1}}^{f_{0}}\epsilon^{1-\alpha }\right]-1\right)f_{0}\left(x\right)dx\leq O\left(\epsilon^{1-\alpha }\right)\max_{x\in M}\left|B(x)\right|$$

## 6. Conditions on Dynamics and Probability Density

As observed in [27], the dissipation function can be obtained starting from the Liouville equation:

(119) $$\frac{\partial f_{t}}{\partial t}\left(x\right)=-f_{t}\left(x\right)\Lambda \left(x\right)-V\left(x\right)\cdot \nabla_{x}f_{t}\left(x\right)$$

and factorizing $f_{t}\left(x\right)$ in the right hand side. The result is Equation (2) with $\Omega^{f_{t}}\left(x\right)$ defined as in (3). The standard conditions of physical interest, under which this operation can be performed are the following:

- the vector field should be everywhere differentiable in the phase space M for Λ to exist;
- the initial density $f_{0}$ should be everywhere positive in M for its logarithm to exist;
- the initial density $f_{0}$ should be everywhere differentiable in M for its gradient to exist.

This also guarantees that the dissipation function be continuous, which is what is used to derive Equation (8) from Equation (7), via Equation (5). In the physical literature, these requirements are satisfied if (a) the condition sometimes called ergodic consistency holds [30]—$f_{0}$ should be the equilibrium ensemble for the dynamics subjected to the same constraints of the nonequilbrium dynamics—and if (b) the particles interactions are sufficiently regular. The effects of discontinuities can be controlled if the dynamics are piecewise regular (as in billiards), or the probability decays sufficiently fast when approaching singularities. A full mathematical treatment of these aspects is far from trivial for physically relevant particle systems, and the success of the theory can only be judged a posteriori, on a case by case basis.

At the same time, various mathematical aspects can be understood considering simple examples, that can be explicitly solved. Let us focus on simple cases in which the above conditions are violated. This is important both to delineate the range of applicability of the theory based on the dissipation function and, at the same time, to find corrections where needed.

When $f_{t}\left(x\right)$ is not differentiable, one may replace (119) by its weak form, i.e.,

(120) $$\int_{M}h\left(x\right)f_{t}\left(x\right)dx=\int_{M}\;h\left(x\right)f_{0}\left(x\right)dx+\int_{0}^{t}\;\int_{M}\;V\left(x\right)\cdot \nabla h\left(x\right)f_{\tau }\left(x\right)dxd\tau \;,\;\mathrm{for}\;\mathrm{all}\;\;h\left(x\right)\in C_{0}^{\infty }\left(M\right),$$

$C_{0}^{\infty }\left(M\right)$ being the set of smooth functions with compact support in M. The non-differentiability, of $f_{t}$, however, is not the only issue. In what follows we take $f_{0}$ and $f_{t}$ with compact support $K_{0}$ and $K_{t}$ in M, while M is not necessarily compact.

### Examples

**Example** **3.** *Consider the dynamical system*

(121) $$\dot{x}=-x\;;\;x\in R$$

*and the following initial density:*

(122) $$f_{0}\left(x\right)=\left\{\begin{array}{cc} 1/2\;, & x\in [-1,1]\\ 0\;, & else \end{array}\right.$$

*that is compactly supported and non differentiable at 1 and −1. As the initial condition x evolves like $S^{t}x=e^{-t}x$, $f_{t}$ is given by*

(123) $$f_{t}\left(x\right)=\frac{1}{2}\;e^{t}\;\chi_{\left[-e^{-t},e^{-t}\right]}\left(x\right)\;,\;with\;\;\chi_{\left[-e^{-t},e^{-t}\right]}\left(x\right)=\left\{\begin{array}{cc} 1 & if\;x\in [-e^{-t},e^{-t}]\\ 0 & if\;x\notin [-e^{-t},e^{-t}] \end{array}\right.$$

Then, Equation (119) does not apply, while Equation (120) does. Indeed, the left hand side and the first term in the right hand side of (120) are

(124) $$A:=\int_{M}h\left(x\right)f_{t}\left(x\right)dx=\frac{e^{t}}{2}\int_{-e^{-t}}^{e^{-t}}\;h\left(x\right)dx\;,\;\mathrm{and}\;B:=\int_{M}\;h\left(x\right)f_{0}\left(x\right)dx=\frac{1}{2}\int_{-1}^{1}h\left(x\right)dx\;.$$

The phase integral in the right hand side of (120) yields:

(125) $$\int_{M}\;V\left(x\right)\cdot \nabla h\left(x\right)f_{s}\left(x\right)dx=-\frac{e^{s}}{2}\int_{-e^{-s}}^{e^{-s}}\;xh^{\prime }\left(x\right)dx=-\frac{1}{2}\left[h\left(e^{-s}\right)+h\left(-e^{-s}\right)\right]+\frac{e^{s}}{2}\int_{-e^{-s}}^{e^{-s}}\;h\left(x\right)dx,$$

and the corresponding time integral becomes:

(126) $$C:=\int_{0}^{t}ds\int_{M}dxV\left(x\right)\cdot \nabla h\left(x\right)f_{s}\left(x\right)=-\frac{1}{2}\int_{0}^{t}\left[h\left(e^{-s}\right)+h\left(-e^{-s}\right)\right]ds+\int_{0}^{t}ds\left[\frac{e^{s}}{2}\int_{-e^{-s}}^{e^{-s}}\;h\left(x\right)dx\right],$$

Then, integrating by parts and using Leibniz’s formula in the last integral, we get:

(127) $$\int_{0}^{t}ds\left[\frac{e^{s}}{2}\int_{-e^{-s}}^{e^{-s}}\;h\left(x\right)dx\right]=\frac{e^{t}}{2}\int_{-e^{-t}}^{e^{-t}}\;h\left(x\right)dx-\frac{1}{2}\int_{-1}^{1}h\left(x\right)dx+\frac{1}{2}\int_{0}^{t}\left[h\left(e^{-s}\right)+h\left(-e^{-s}\right)\right]ds,$$

and

(128) $$C=\frac{e^{t}}{2}\int_{-e^{-t}}^{e^{-t}}\;h\left(x\right)dx-\frac{1}{2}\int_{-1}^{1}h\left(x\right)dx.$$

Form (124) we get A=B+C, that proves (120).

The probability with density $f_{t}\left(x\right)$ converges weakly to the delta function at x=0, since for any continuous O, the mean value theorem yields:

$${\langle O\rangle }_{t}=\int O\left(x\right)f_{t}\left(x\right)dx=\frac{e^{t}}{2}\int_{-e^{-t}}^{e^{-t}}\;O\left(x\right)dx=O\left(x_{t}\right)\to O\left(0\right)\;\mathrm{as}\;t\to +\infty \;,$$

for one $x_{t}\in \left(-e^{-t},e^{-t}\right)$. Can this result be obtained via the response formula (8)? Because Λ(x)=−1, and $df_{0}/dx=0$, the dissipation function takes the form:

(129) $$\Omega^{f_{0}}\left(x\right)=-\Lambda \left(x\right)-V\left(x\right)\;\frac{f_{0}^{\prime }\left(x\right)}{f_{0}\left(x\right)}=1$$

on the interval [−1,1]. Hence, unlike the cases addressed so far, ${\langle \Omega^{f_{0}}\rangle }_{0}$ does not vanish, it equals 1!

More generally, given the 1-D system $\dot{x}=V\left(x\right)$ with x∈R and $f_{0}$ supported on any interval [a,b], integration by parts yields:

(130) $$\begin{array}{c} {\langle \Omega^{f_{0}}\rangle }_{0}=\int_{a}^{b}\left(-V^{\prime }\left(x\right)-V\left(x\right)\;\frac{f_{0}^{\prime }\left(x\right)}{f_{0}\left(x\right)}\right)f_{0}\left(x\right)dx=V\left(a\right)f_{0}\left(a\right)-V\left(b\right)f_{0}\left(b\right), \end{array}$$

showing that ${\langle \Omega^{f_{0}}\rangle }_{0}$ vanishes only if some conditions, such as V(a)=V(b)=0 or $f_{0}\left(a\right)=f_{0}\left(b\right)=0$, are verified.

In Ref. [27], the equality ${\langle \Omega^{f_{t}}\rangle }_{f_{t}}=0$ was obtained from the conservation of probability and from the interchange of the time derivative with the integration in phase space:

(131) $${\langle \Omega^{f_{t}}\rangle }_{f_{t}}=\int_{M}\Omega^{f_{t}}f_{t}d\Gamma =\int_{M}\frac{\partial f_{t}}{\partial t}d\Gamma =\frac{d}{dt}\int_{M}f_{t}d\Gamma =\frac{d}{dt}1=0$$

This exchange is legitimate under the joint continuity w.r.t. *x* and *t* of $f_{t}$ and of $\partial f_{t}/\partial t$, which is not the case of our example. Consequently, Equation (8) leads to the nonsensical expression:

(132) $${\langle O\rangle }_{t}={\langle O\rangle }_{0}+\int_{0}^{t}d\tau \int_{-1}^{1}O\left(e^{-t}x\right)dx\;,$$

which diverges linearly with *t*, rather than converging to O(0), because $O\left(e^{-t}x\right)\to O\left(0\right)$. This is related to the fact that ${\langle \Omega^{f_{0}}\rangle }_{0}\neq 0$, due to the non differentiability of $f_{0}$. If, on the other hand, one has $f_{0}\left(x\right)=3\left(1-x^{2}\right)/4$ with x∈[−1,1], one obtains:

(133) $$\Omega^{f_{0}}\left(x\right)=\frac{1-3x^{2}}{1-x^{2}}\;,\;\mathrm{which}\mathrm{leads}\mathrm{to}\;\;{\langle \Omega^{f_{0}}\rangle }_{0}=0$$

Then, taking for instance $O\left(x\right)=x^{n}$, which can be used for analytic functions, one has:

(134) $${\langle O\rangle }_{t}={\langle O\rangle }_{0}+\frac{3}{4}\int_{0}^{t}ds\;e^{-ns}\int_{-1}^{1}dx\;x^{n}\left(1-3x^{2}\right)=\frac{3}{\left(n+1)(n+3\right)}+\frac{3}{\left(n+1)(n+3\right)}\left[e^{-nt}-1\right]$$

for even *n*, which correctly converges to O(0)=0, and 0 for odd *n*, which equals O(0) at all times.

The role of Equation (13) is further clarified noticing that for a finite *t* one can write:

(135) $$\begin{array}{cc} \frac{1}{t}\int_{0}^{t}{\langle \left(O\circ S^{\tau }\right)\cdot \Omega^{f_{0}}\rangle }_{0}d\tau & =\frac{1}{t}\int_{0}^{t}d\tau \int_{M}\left(O\circ S^{\tau }\left(x\right)\right)\cdot \Omega^{f_{0}}\left(x\right)f_{0}\left(x\right)dx\\ \; & =\int_{M}\left[\frac{1}{t}\int_{0}^{t}\left(O\circ S^{\tau }\left(x\right)\right)d\tau \right]\Omega^{f_{0}}\left(x\right)f_{0}\left(x\right)dx \end{array}$$

Then, assuming the t→∞ limit and the phase integration can be exchanged in Equation (135), the previous equation yields:

$$\lim_{t\to \infty }\frac{1}{t}\int_{0}^{t}{\langle \left(O\circ S^{\tau }\right)\cdot \Omega^{f_{0}}\rangle }_{0}d\tau =\bar{O}\cdot {\langle \Omega^{f_{0}}\rangle }_{0}$$

where $\bar{O}$ is the time average of O. This shows that Equation (9) requires ${\langle \Omega^{f_{0}}\rangle }_{0}=0$, i.e., that either $\Omega^{f_{0}}$ vanishes or changes sign in M.

On the other hand, one realizes that this is not sufficient in general. For instance, despite the correct result (134), the blind application of Equation (5) for the case of Equation (121) with density $3(1-x^{2})/4$ in [−1,1], yields an incorrect evolved density $f_{t}$. In this case, we have:

(136) $$\Omega^{f_{0}}\left(S^{s}x\right)=\Omega^{f_{0}}\left(e^{-s}x\right)=\frac{e^{2s}-3x^{2}}{e^{2s}-x^{2}}$$

and

(137) $$\Omega_{-t,0}^{f_{0}}\left(x\right)=\int_{-t}^{0}\Omega^{f_{0}}\left(S^{s}x\right)ds=3t+\ln\frac{\left|e^{-2t}-x^{2}\right|}{1-x^{2}}$$

which implies:

(138) $$f_{t}\left(x\right)=\frac{3}{4}\left|e^{-2t}-x^{2}\right|e^{3t}$$

that is not normalized on [−1,1]. This difficulty is not related to the violation of Equation (13), but to the fact that the support of $f_{t}$ changes with time or, equivalently, that Equation (5) attributes a positive probability to points outside [−1,1]. An analogous, even more macroscopic, situation arises if M=[1,2] and $f_{0}\left(x\right)=2\left(3-x\right)/3$, which yields $V\left(1\right)f_{0}\left(1\right)=V\left(2\right)f_{0}\left(2\right)$, hence yields (13) with non vanishing V(1),V(2) and $f_{0}\left(1\right),f_{0}\left(2\right)$. This stresses that dynamical equations and phase space must be properly chosen.

The point is that the preimages $S^{-s}x$ of part of the points in M, for some s>0, lie outside M. In these cases, Equation (5) requires that only the contributions of the points *x* with $S^{-s}x\in M$ be included in the calculations, as discussed in Ref. [32].

**Example** **4.** *Consider the dynamical system*

(139) $$\dot{x}=x-x^{3},$$

*that has two asymptotically stable fixed points $x_{-}=-1$ and $x_{+}=1$, and an unstable fixed point, $x_{0}=0$. Take the invariant set M=[−1,1] as the phase space of the system. The flow is given by:*

(140) $$S^{t}x=\frac{xe^{-t}}{\sqrt{1+x^{2}\left(e^{-2t}-1\right)}}\;,\;x\in M.$$

Assuming $f_{0}\left(x\right)=1/2$, uniform on M, the dissipation function is expressed by: $\Omega^{f_{0}}\left(x\right)=-\Lambda \left(x\right)=3x^{2}-1$, hence it changes sign in M. The evolved distribution is given by:

(141) $$f_{t}\left(x\right)=\frac{1}{2}\chi_{\left[-1,1\right]}\left(x\right)\;e^{-t}\;{\left(1-x^{2}+x^{2}e^{-2t}\right)}^{-\frac{3}{2}}\;.$$

If, on the other hand, $f_{0}\left(x\right)=\frac{3}{4}\left(1-x^{2}\right)$, we have

(142) $$\Omega^{f_{0}}\left(x\right)=5x^{2}-1$$

that also changes sign, and the density at time *t* becomes:

(143) $$f_{t}\left(x\right)=\frac{3}{4}e^{-t}\;\left(1-x^{2}\right)\;{\left(1-x^{2}+x^{2}e^{-2t}\right)}^{-\frac{5}{2}}\;.$$

From (141) and (143) one can see that in both cases $d\mu_{t}=f_{t}\left(x\right)dx$ converges weakly to $\frac{1}{2}\left(\delta_{-1}+\delta_{1}\right)$. This is correctly so because the flow vanishes at the boundaries of the phase space: M is invariant; hence the points inside M have no preimage outside M. In this case, the non-differentiability of the uniform density in R has no bearing on the theory.

**Example** **5.** *Let us consider the one-dimensional dynamical system:*

(144) $$\dot{x}=-x+\epsilon x^{3},\;0<\epsilon \leq 1,$$

*and denote $V_{0}\left(x\right)=-x$ and $V_{p}\left(x\right)=x^{3}$. This system has three fixed points: $x_{0}=0$, which is asymptotically stable, and the unstable points $x_{-}=-1/\sqrt{\epsilon }$ and $x_{+}=1/\sqrt{\epsilon }$. We denote by $M_{\epsilon }$ the set $\left[-1/\sqrt{\epsilon },1/\sqrt{\epsilon }\right]⊃\left[-1,1\right]$, which is invariant, being invariant the intervals $\left[-1/\sqrt{\epsilon },0\right]$ and $\left[0,1/\sqrt{\epsilon }\right]$. Integrating the differential equation, we obtain*

(145) $$S_{\epsilon }^{t}x=\frac{xe^{-t}}{\sqrt{1+\epsilon x^{2}\left(e^{-2t}-1\right)}},\;for\;x\in M_{\epsilon }.$$

Then, expanding with respect to ϵ,

(146) $$S_{\epsilon }^{t}x=S_{0}^{t}x-\frac{\epsilon }{2}x^{3}e^{-t}\left(e^{-2t}-1\right)+O\left(\epsilon^{2}x^{5}e^{-t}{\left(e^{-2t}-1\right)}^{2})\right),\;x\in M_{\epsilon },\;t\geq 0,\;0\leq \epsilon \leq 1,$$

where $S_{0}^{t}x=xe^{-t}$. The uniform probability on [−1,1]$f_{0}\left(x\right)=1/2$, has compact support in $M_{\epsilon }⊃\left[-1,1\right]$. Substituting $\Omega_{V_{0}}^{f_{0}}\left(x\right)=1$ and $\Omega_{V_{p}}^{f_{0}}\left(x\right)=-3x^{2}$ in Equation (47) yields:

(147) $$\begin{array}{cc} f_{t}\left(x;\epsilon \right) & =\frac{1}{2}\chi_{\left[-1,1\right]}\left(x\right)\exp\left[\int_{-t}^{0}d\tau \right]\exp\left[-3\epsilon \int_{-t}^{0}\frac{x^{2}e^{-2\tau }}{1+\epsilon x^{2}\left(e^{-2\tau }-1\right)}d\tau \right]\\ \; & =\frac{1}{2}\chi_{\left[-1,1\right]}\left(x\right)\;e^{t}\;{\left(1-\epsilon x^{2}+\epsilon x^{2}e^{2t}\right)}^{-\frac{3}{2}}. \end{array}$$

This allows us to check that the probability measure $d\mu_{t}^{\epsilon }\left(x\right)=f_{t}\left(x;\epsilon \right)\;dx$ converges weakly to the delta function at x=0 as t→∞ The linear approximation for small ϵ can be obtained, in analogy with the Hamiltonian case, cf. (54), by truncating the exponential in (47):

(148) $$\begin{array}{cc} {\tilde{f}}_{t}\left(x;\epsilon \right) & =f_{0}\left(x\right)\exp\left[\int_{0}^{t}d\tau \;\Omega_{V_{0}}^{f_{0}}\left(S_{\epsilon }^{-\tau }x\right)\right]\left[1+\epsilon \int_{0}^{t}d\tau \;\Omega_{V_{p}}^{f_{0}}\left(S_{\epsilon }^{-\tau }x\right)\right] \end{array}$$

which gives:

(149) $${\tilde{f}}_{t}\left(x;\epsilon \right)=\frac{1}{2}\chi_{\left[-1,1\right]}\left(x\right)e^{t}\left[1-\frac{3}{2}\ln\left(1-\epsilon x^{2}+\epsilon x^{2}e^{2t}\right)\right].$$

It can also be observed that the linear approximation at ϵ=0 of the exact evolved density (147),

(150) $${\hat{f}}_{t}\left(x;\epsilon \right)=\frac{1}{2}\chi_{\left[-1,1\right]}\left(x\right)\;e^{t}\;\left[1-\frac{3}{2}\left(1-\epsilon x^{2}+\epsilon x^{2}e^{2t}\right)\right],$$

does not coincide with Equation (149), but it can be obtained from Equation (149) approximating lnx to first order in *x*.

Given that $\Omega_{V_{0}+\epsilon V_{p}}^{f_{0}}=-\left(1-3\epsilon x^{2}\right)$, the exact response of ensemble averages is given by:

(151) $${\langle O\rangle }_{t}={\langle O\rangle }_{0}-\frac{1}{2}\int_{0}^{t}d\tau \int_{-1}^{1}O\left(S_{\epsilon }^{\tau }x\right)\left(1-3\epsilon x^{2}\right)dx,$$

that, on the basis of the previous remarks, converges to O(0), as t→∞. As observed in Section 3, the linear approximation analogous to the GK response is obtained by replacing the exact flow $S_{\epsilon }^{t}x$ with the linearized one, $S_{0}^{t}x=x\;e^{-t}$, in Equation (151).

Now, let *A* and *B* be smooth functions, and $f_{0}$ a probability density with compact support *K* in $M_{\epsilon }$, containing x=0 in its interior, for instance K=[−1,1]. In order to check *T*-mixing, see (11), let us write:

(152) $$\begin{array}{cc} & \;\lim_{t\to \infty }\left[\int_{-1}^{1}A\left(S_{\epsilon }^{t}x\right)B\left(x\right)f_{0}\left(x\right)dx-\int_{-1}^{1}A\left(S_{\epsilon }^{t}x\right)f_{0}\left(x\right)dx\int_{-1}^{1}B\left(x\right)f_{0}\left(x\right)dx\right] \end{array}$$

(153) $$\begin{array}{c} \;=A\left(0\right)\int_{-1}^{1}B\left(x\right)f_{0}\left(x\right)dx-A\left(0\right)\int_{-1}^{1}B\left(x\right)f_{0}\left(x\right)dx=0 \end{array}$$

which follows from the uniform convergence of $S_{\epsilon }^{t}x$ to 0, from the boundedness of *A* and *B* in any compact set and from the continuity of *A*. This proves the T-mixing property (11) for the present example. The same reasoning shows that the system is also ΩT-mixing.

**Example** **6.** *Consider the one-dimensional dynamical system:*

(154) $$\dot{x}=x-\epsilon x^{3},\;0\leq \epsilon \leq 1,$$

*that is the time reversed version of (144). The point $x_{0}=0$ is now unstable, while $x_{-}=-1/\sqrt{\epsilon }$ and $x_{+}=1/\sqrt{\epsilon }$ are stable, and $M_{\epsilon }=\left[-1/\sqrt{\epsilon },1/\sqrt{\epsilon }\right]⊃\left[-1,1\right]$ is invariant. The flow is expressed by:*

(155) $$S_{\epsilon }^{t}x=\frac{xe^{t}}{\sqrt{1+\epsilon x^{2}\left(e^{2t}-1\right)}}=S_{0}^{t}x-\frac{\epsilon }{2}x^{3}e^{t}\left(e^{2t}-1\right)+O\left(\epsilon^{2}x^{5}e^{t}{\left(e^{2t}-1\right)}^{2})\right),\;for\;x\in M_{\epsilon }.$$

*where $S_{0}^{t}x=xe^{t}$. The ϵ expansion at ϵ=0*

(156) $$S_{\epsilon }^{t}x=S_{0}^{t}x-\frac{\epsilon }{2}x^{3}e^{t}\left(e^{2t}-1\right)+...$$

*has an error that grows without limit as t increases, which shows the unsuitability of the linear response. In this case, always assuming $f_{0}\left(x\right)=1/2$ in [−1,1], Equation (47) yields*

(157) $$\begin{array}{cc} f_{t}\left(x;\epsilon \right) & =\frac{1}{2}\chi_{\left[-1,1\right]}\left(x\right)\exp\left[-\int_{-t}^{0}d\tau \right]\exp\left[3\epsilon \int_{-t}^{0}\frac{x^{2}e^{2\tau }}{1+\epsilon x^{2}\left(e^{2\tau }-1\right)}d\tau \right]\\ \; & =\frac{1}{2}\chi_{\left[-1,1\right]}\left(x\right)\;e^{-t}\;{\left(1-\epsilon x^{2}+\epsilon x^{2}e^{-2t}\right)}^{-\frac{3}{2}}, \end{array}$$

*being $\Omega_{V_{0}}^{f_{0}}\left(x\right)=-1$ and $\Omega_{V_{p}}^{f_{0}}\left(x\right)=3x^{2}$, since $V_{0}\left(x\right)=x$ and $V_{p}\left(x\right)=-x^{3}$. Observe that $f_{t}\left(\pm 1/\sqrt{\epsilon };\epsilon \right)\to \infty$ while $f_{t}\left(x;\epsilon \right)\to 0$ for $-1/\sqrt{\epsilon }<x<1/\sqrt{\epsilon }$ as t→∞. Thus the measure $d\mu_{t}^{\epsilon }\left(x\right)=f_{t}\left(x;\epsilon \right)\;dx$ converges weakly to $\frac{1}{2}\delta_{-1/\sqrt{\epsilon }}+\frac{1}{2}\delta_{1/\sqrt{\epsilon }}$.*

**Example** **7.** *Let us consider*

(158) $$\dot{x}=\epsilon \left(x-x^{3}\right),\;-1\leq \epsilon \leq 1,$$

*and assume the invariant interval M=[−1,1] as the phase space. For ϵ≠0 the fixed points are $x_{0}=0$, $x_{-}=-1$ and $x_{+}=1$. For ϵ>0, the point 0 is unstable, while −1 and 1 are stable. The opposite holds for ϵ<0. For ϵ=0, all the points are fixed. The flow is given by:*

(159) $$S_{\epsilon }^{t}x=\frac{xe^{\epsilon \;t}}{\sqrt{1+\epsilon x^{2}\left(e^{2\epsilon \;t}-1\right)}}.$$

*Taking the initial uniform distribution on [−1,1], being $\Omega_{V}^{f_{0}}=-\epsilon \left(1-3x^{2}\right)$, the evolved density is:*

(160) $$\begin{array}{c} f_{t}\left(x;\epsilon \right)=\frac{1}{2}\chi_{\left[-1,1\right]}\left(x\right)\;e^{-\epsilon \;t}\;{\left(1-\epsilon x^{2}+\epsilon x^{2}e^{-2\epsilon t}\right)}^{-\frac{3}{2}}. \end{array}$$

*For ϵ>0 the distribution $d\mu_{t}^{\epsilon }\left(x\right)=f_{t}\left(x;\epsilon \right)\;dx$ converges to $\frac{1}{2}\delta_{-1}+\frac{1}{2}\delta_{1}$, while for ϵ<0 the limit is $\delta_{0}$. The response is*

(161) $${\langle O\rangle }_{t}={\langle O\rangle }_{0}-\frac{\epsilon }{2}\int_{0}^{t}d\tau \int_{-1}^{1}O\left(S_{\epsilon }^{\tau }x\right)\left(1-3\epsilon x^{2}\right)dx$$

*Once again, the linear response approach is scarcely interesting.*

## 7. Concluding Remarks

In this paper we have reviewed the developments that led from nonequilbrium molecular dynamics (NEMD) to the fluctuation relations (FR) and to the exact response theory, which develops the previous formalism based on the so-called Transient Time Correlation Function [6]. Within this framework, some authors have adopted the phase space contraction rate −Λ as a definition of the steady state entropy production rate σ [23,41], after −Λ and σ had been observed to be proportional to each other, in the original paper on fluctuations relations [36]. That paper dealt with the Gaussian thermostatted iso-energetic model of a shearing fluid known as SLLOD. In that case, the steady phase space contraction and entropy production rates could indeed be directly related to each other, something that in NEMD models had already been pointed earlier [19,51,52]. Mostly the interest had been on steady state state averages of such quantities. It was then realized that such an identification is justified only for steady state averages of certain NEMD models and that, in general, fluctuations require −Λ to be distinguished from the energy dissipation rate [24,25,53]. At the same time, the function that represents energy dissipation in molecular dynamics has been identified as the dissipation function Ω, the function that obeys the FR. For the transient FR this happens quite generally; for the steady state FR it is required that correlations w.r.t. the initial distribution do not grow too fast, something implied by the T-mixing condition [26].

Through a number of examples, including NEMD models as well as simple exactly solvable models, we have illustrated these facts and how the exact response formula, based on Ω, applies. In particular we have analyzed the relation between Ω and −Λ, noting that in the analysis of generic dynamical systems the two quantities are by and large equivalent, while in the case of particle systems the first corresponds to a real observable. We have then compared the exact response formula with the one of the Green-Kubo linear theory, quantifying their difference, that typically grows with the size of the perturbation and of time, but also showing that in some simple case they yield the same result.

We have then investigated “formal thermodynamics’’ along the lines of Ref. [29], introducing a formal temperature and a formal energy flow, and using Ω instead of −Λ as a notion of formal entropy production rate. Analogously to earlier works, e.g., Refs. [54,55,56], this means that the phase space is treated as real space, and its different regions as different locations in real space. Clearly, the 2dN-dimensional phase space of an *N* particle system in *d* dimensions and the phase points that flow in it are totally different from the 2d-dimensional real space and the particles that flow through it [43,44]. (A point in phase space has no extension nor “inertia’’, it does not interact with other points, and it equally represents the microstate of a large or small system. Any other point represents another system that can be arbitrarily close or far from it. A particle in real space has a finite extension, interacts with nearby particles, and follows Newton’s laws with its inertia. Just this has numerous consequences. For instance, while the Gibbs entropy represents thermodynamic entropy only at equilibrium, the Boltzmann entropy works also in time dependent situations, Refs. [44,57]). Therefore, in general, a direct identification of flows and related quantities in phase space with their physical counterparts is not possible. Nevertheless, this analysis is reminiscent of the thermodynamic formalism, which is important for dynamical systems in general [33]. Furthermore, it may find direct physical application in systems of non-interacting particles, for which the factorization of the phase space distributions makes phase space and real space almost the same. Such systems may not behave thermodynamically, but they are of greater and greater interest in e.g., bio- and nano-technology (cf. transport in highly confining media), for photons in optical wave-guides or random media, for cold atoms etc.

Finally, we have observed that the exact response formula (8), mainly rests on the regularity of the vector fields, and on the ergodic consistency of the initial distribution, which ensure the regularity of the dissipation function as well. These mathematical conditions need further scrutiny, in more realistic cases, but are relatively minimal, hence hint at a wide applicability of the theory. The investigation of physical applications of this theory is just at the beginning; many are indeed the cases in which perturbations cannot be taken as small compared to the reference signals; they range from nano-technology to geophysical and even astrophysical phenomena [58].
